# Advances in fundamentals and application of plasmon-assisted CO_2_ photoreduction

**DOI:** 10.1515/nanoph-2023-0793

**Published:** 2024-02-01

**Authors:** Zelio Fusco, Fiona J. Beck

**Affiliations:** School of Engineering, College of Engineering and Computer Science, Australian National University, Canberra, ACT 2601, Australia

**Keywords:** plasmonic, photocatalysis, hot carriers, CO_2_ reduction

## Abstract

Artificial photosynthesis of hydrocarbons from carbon dioxide (CO_2_) has the potential to provide renewable fuels at the scale needed to meet global decarbonization targets. However, CO_2_ is a notoriously inert molecule and converting it to energy dense hydrocarbons is a complex, multistep process, which can proceed through several intermediates. Recently, the ability of plasmonic nanoparticles to steer the reaction down specific pathways and enhance both reaction rate and selectivity has garnered significant attention due to its potential for sustainable energy production and environmental mitigation. The plasmonic excitation of strong and confined optical near-fields, energetic hot carriers and localized heating can be harnessed to control or enhance chemical reaction pathways. However, despite many seminal contributions, the anticipated transformative impact of plasmonics in selective CO_2_ photocatalysis has yet to materialize in practical applications. This is due to the lack of a complete theoretical framework on the plasmonic action mechanisms, as well as the challenge of finding efficient materials with high scalability potential. In this review, we aim to provide a comprehensive and critical discussion on recent advancements in plasmon-enhanced CO_2_ photoreduction, highlighting emerging trends and challenges in this field. We delve into the fundamental principles of plasmonics, discussing the seminal works that led to ongoing debates on the reaction mechanism, and we introduce the most recent *ab initio* advances, which could help disentangle these effects. We then synthesize experimental advances and *in situ* measurements on plasmon CO_2_ photoreduction before concluding with our perspective and outlook on the field of plasmon-enhanced photocatalysis.

## 1 Background and motivation

The ever-accelerating pace of industrialization and anthropogenic activities has led to an alarming increase in atmospheric CO_2_ levels over the past 100 years, reaching >420 ppm in 2023 [1], fuelling concerns about climate change and its far-reaching consequences. Addressing this critical environmental challenge demands innovative and multifaceted approaches that not only reduce CO_2_ emissions but also transform them into valuable chemicals and fuels. Particularly, hydrocarbons with more than one carbon atom per molecule (C2_+_) are valuable due to their high energy density, versatility, ease of storage and transportation and large, established market. Unfortunately, producing these chemicals using current methods relying on fossil fuels emits millions of tonnes of CO_2_-equivalent greenhouse gases per year.

Artificial photosynthesis enables a closed-loop solution for producing hydrocarbons from CO_2_ and water using sunlight to drive the reaction. If the CO_2_ is directly captured from the atmosphere, this has the potential to provide renewable fuels at scale with net-zero emissions, without the need for additional electricity inputs. Upon light absorption, photocatalytic materials including semiconductors and metals generate electron–hole pairs, which migrate to the catalyst’s surface and can be used to drive two related redox half reactions: electrons for reducing CO_2_ and holes for oxidizing water [2], [3]. While both sides of the reactions are important for a complete treatment of artificial photosynthesis, the CO_2_ reduction is a more complex and harder to realize.

**Scheme 1:**
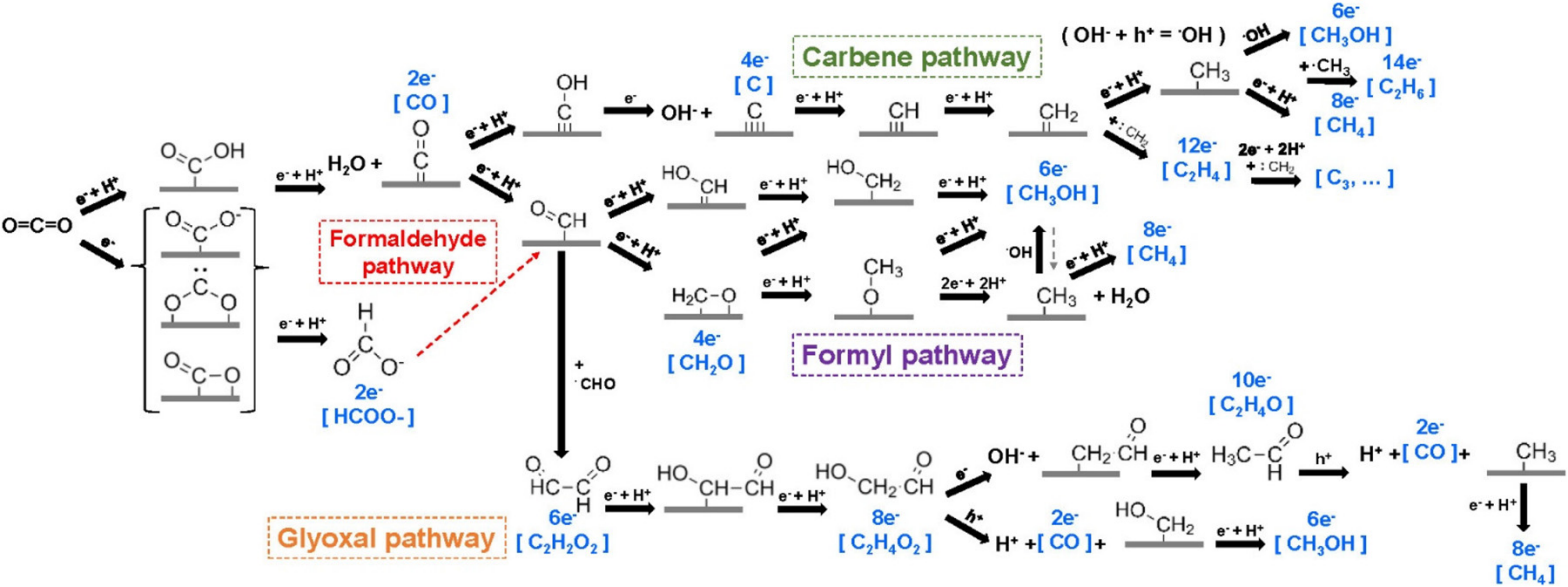
Mechanistic pathways of CO_2_ reduction to commonly observed C_1_ and C_2_ species on metal surfaces. Reproduced with permission from ref [7]. Copyright 2022, American Chemical Society.

Due to its stability, CO_2_ reduction into higher-value products is a complex, multistep process requiring multiple electron and proton transfers. As shown in Scheme 1, the existence of multiple, branching and co-existing reaction pathways can lead to a variety of different hydrocarbons. The challenge in achieving commercially viable renewable production of valuable (C2_+_) hydrocarbon products using solar energy and CO_2_ is selectively producing the desired product with high yields. Ultimately, this depends on the physicochemical properties of the materials and is a critical object of research. For this reason, a large body of recent work has focused exclusively on the rational design of efficient photocatalysts for CO_2_ reduction half reaction, and studies often use hole scavengers as sacrificial electron donors, to prevent surface hole accumulation and improve charge separation [2], [4], [5], [6].

Leveraging the exquisite control of light–matter interactions at the nanoscale afforded by plasmonic nanostructures offers a prospective avenue for efficiently facilitating chemical transformations. Recently, the use of plasmonic materials as active elements has sparked renewed interest the field of plasmonics, thanks to the demonstration of enhanced photochemical reaction and the promise of achieving reaction selectivity [4], [8], [9], [10], [11], [12], [13], suggesting a strategy for sustainable chemical production.

Upon light interaction, nanostructured metals exhibit strong light–matter interactions, resulting in the excitation of localized surface plasmon resonances (LSPRs). These provide a way to concentrate light in subwavelength volumes resulting in highly enhanced electric fields strongly confined at the nanostructure’s surface. Following their excitation, the non-radiative decay of the LSPRs generates a population of highly energetic electron–hole pairs, commonly referred to as hot carriers. These photo-excited carriers gradually lose their energy through scattering events and ultimately release it to the environment as heat, resulting in a localized temperature increase. Enhanced fields, hot carriers and thermal gradient – either individually or synergistically – can be harnessed to drive and promote chemical reactions [14], [15], [16], [17], [18], [19]. Nonetheless, despite seminal contributions and proof-of-concept demonstrations, the anticipated transformative impact of plasmonics on the field of photocatalysis has yet to materialize in practical applications.

In this review, we will elucidate the challenges and address the opportunities in realizing practical applications of plasmon-mediated CO_2_ photocatalysis. We aim to provide a comprehensive overview of the recent advancements in plasmonic photocatalysis for CO_2_ reduction, focusing on the key mechanisms, challenges and prospects. For a thorough exploration of the other half-reaction and an in-depth discussion on the role of holes in chemical reactions, we refer the readers to excellent recent reviews on the topic [20], [21], [22]. We will start by discussing the fundamentals of plasmon photocatalysis and the seminal works that have led to the ongoing debate on the reaction mechanism. In doing so, we will clarify the multiple adopted definitions and nomenclature. We will then discuss the most recent theoretical advances in understanding the opto-electronic properties of plasmonic materials and how they interface with the catalysis field, paying particular attention to their coupling and interactions with CO_2_. Here, we will introduce *ab initio* density functional theory calculations, along with their challenges and perspectives. Next, we will discuss recent experimental demonstrations of plasmonic CO_2_ photoreduction, differentiating between *in situ* measurement, often used for investigation of the plasmon action mechanisms, and *ensemble* measurements, aimed at demonstrating high reaction yields, efficiency and selectivity. Here, we will analyse how the theoretical results discussed in the previous section support and/or contrast the experimental findings. Finally, we will provide our point of view on the development of this field for scalable renewable fuel production from CO_2_ splitting. We will conclude by discussing the challenges and opportunities to realize the practical application of plasmonic photocatalysis for large scale renewable fuel production.

### 1.1 Plasmon excitation and relaxation

In the past few decades, the rapid development of nanotechnology has enabled subwavelength structuring of materials, allowing the control of light–matter interactions at optical wavelengths [23], [24], [25], [26], [27]. Particular attention has been given to metallic nanoparticles, due to their ability to strongly interact and couple with photons of suitable energy, their ease of fabrication and the ability to easily model their optical behaviour [28], [29], [30].

**Figure 1:**
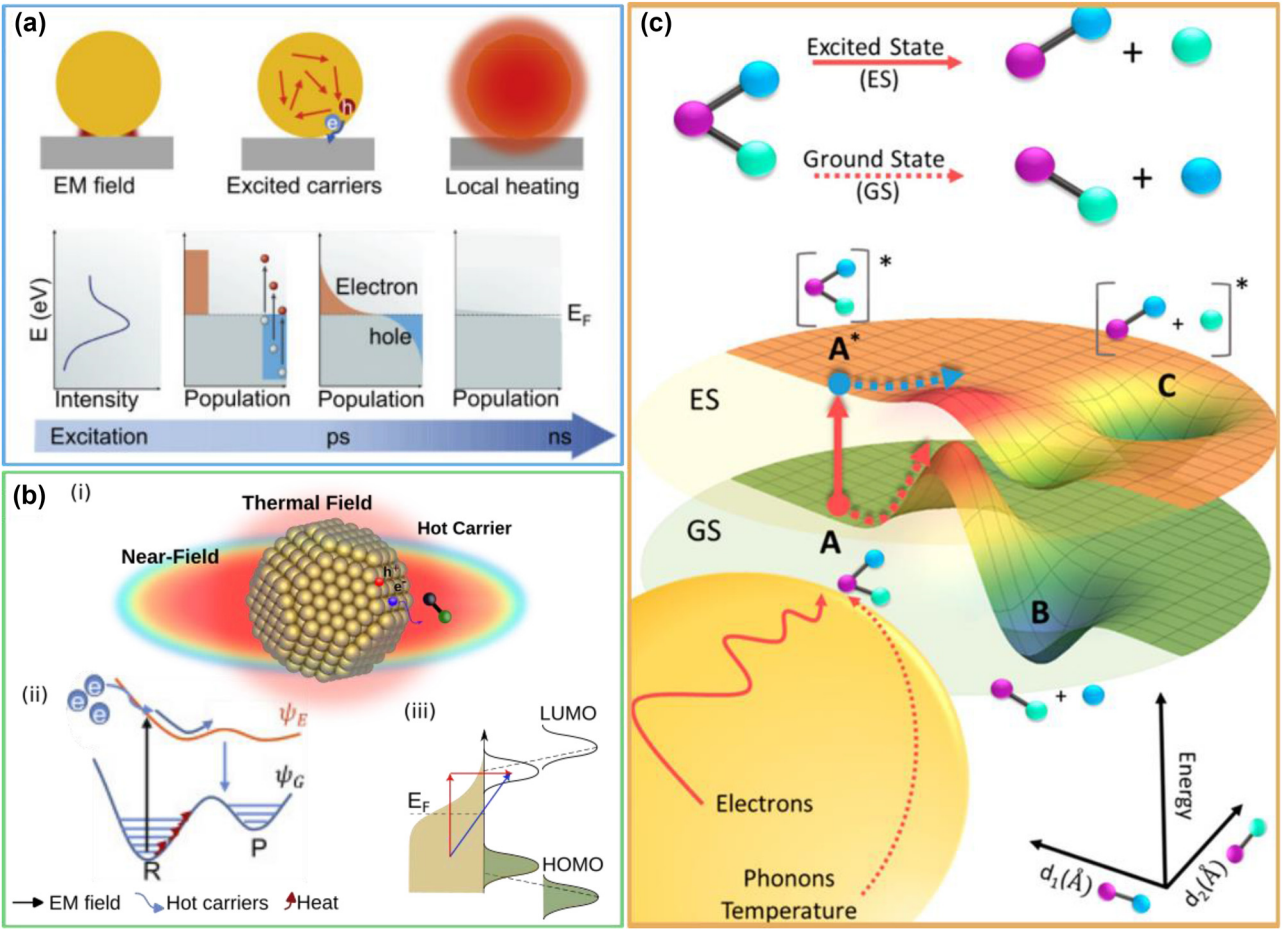
Plasmon relaxation process and energy transfer mechanisms. (a) Main effects resulting from plasmon excitation and relaxation schematically represented in the space, energy and time domains. These are classified in non-thermal (electromagnetic near-fields and hot carriers) and thermal effects (local heating). (b-i) Schematic of the possible plasmon energy transfer mechanisms in the space domain. (b-ii) Plasmon action mechanisms as a function of the energy and reaction coordinate. *R* and *P* are the reactant and product molecules, respectively, while *φ*_G_ and *φ*_E_ represents the ground and an excited state PESs, respectively. Near-fields and hot carriers can promote the system to an excited PES, while local heat can provide only vibrational energy along *φ*_G_. (b-iii) Hybridization between molecular and metallic states can decrease the energy gap required for transferring hot electrons. These can be transferred directly (blue arrow) or indirectly (red arrows). (c) Rendering of plasmon-enabled selectivity via promotion of the system from the ground to an excited potential energy surface. The excited state may have lower activation barrier for product generation (higher efficiency) and different minima (linked to selectivity). (a) Reproduced with permission from [14]. Copyright 2020, Elsevier. (b-i, b-iii) Adapted with permission from [17]. Copyright 2022, The Royal Society of Chemistry. (b-ii) Reproduced with permission from [14]. Reproduced with permission from [14]. Copyright 2020, Elsevier. (c) Reproduced with permission from [18]. Copyright 2019, American Chemical Society.

Within illuminated metal nanostructures, the alternating electric component of incident electromagnetic waves induces a coherent displacement of the metal free electron density, which is counterbalanced by a restoring coulombic force between the nuclei and the electrons. This results in the collective oscillations of the metal conduction electrons, known as localized surface plasmon resonance (LSPR) [31]. When resonantly excited, metallic nanoparticles (NPs) can interact with incident light over areas much larger than their own geometric cross section and can absorb and concentrate light in subwavelength volumes [32], effectively acting as nanoscale optical antennas [31], [33]. This interaction results in light energy confinement in the form of enhanced electromagnetic fields at the surface of the metal nanostructure [34], [35], which are localized within the first ∼30 nm from the nanoparticle’s surface (Figure 1(a)) [36]. These are commonly referred to as near-fields and are heavily exploited in surface-enhanced spectroscopies [32], [37], [38].

Once excited, plasmons can decay radiatively by photon emission or non-radiatively by generating a population of highly energetic charge carriers. The latter are out-of-equilibrium electron–hole pairs generated within 100 fs with an energy distribution that cannot be described by the Fermi–Dirac statistics and are commonly referred to as hot carriers [39], [40]. Recent theoretical investigation on the spatial distribution of hot carriers within small Ag clusters revealed that hot holes accumulated at atomic sites throughout the particle, while hot electrons reside mainly in the surface region [41]. Utilization of these hot carriers often requires charge separation, which can be achieved for instance by forming a junction at metal–semiconductor interfaces [42], [43], [44], [45]. Within hundreds of fs, the hot carriers are subjected to internal electron–electron scattering events, which results in a gradual redistribution of their energy and establishment of a population of carriers in thermal equilibrium with a well-defined electronic temperature (*T*_e_), higher than that of the lattice, *T*_L_. This process is commonly referred to as thermalization and can be described by using the so-called two-temperature model [46], [47]; at this stage, the energy distribution of the hot carriers can be described by a Fermi–Dirac distribution. Further electron–phonon scattering leads to equilibration between *T*_e_ and *T*_L_ within few picoseconds and results in an increased temperature of the metal [46], [48]. While the photophysical process for hot electron and hot holes generation is the same, it is worth noting that the photo-generated hot holes usually have a shorter lifetime than hot-electrons [41], [49], [50], making them harder to employ in driving redox reactions [20]. Ultimately, within nanoseconds, the heat is transferred to the environment through phonon–phonon interactions, resulting in a macroscopic temperature increase [14], [48]. Figure 1(b) schematically shows these relaxation effects.

### 1.2 Plasmon energy transfer mechanisms

During the excitation and relaxation processes of the LSPRs, three different mechanisms transfer energy from the optical source to surrounding molecular species or semiconductors via the plasmonic nanostructure: enhanced near-fields, hot carriers and heat, as shown in Figure 1(b, i-ii). These transfer processes can be classified into non-thermal and thermal effects, where the former include near-field enhancements and hot carrier generation, while the latter refers to the local heating effects [17], [19], [51], [52]. Some authors have also referred these effects as photochemical and photothermal, respectively [11], [53], [54].

#### 1.2.1 Near-field enhancement

Near-field enhancements can induce optical transitions of adjacent molecules via excitation between highest unoccupied molecular orbital (HOMO) and lowest unoccupied molecular orbital (LUMO) [11], [33], [55], [56], [57], promoting the catalyst–reactant system, (consisting of the metal nanostructured catalysts and an absorbed molecular species), to an excited potential energy surface (PES), as shown in by *ψ*_E_ in Figure 1(b-ii). This only takes place when there is spectral overlap between the LSPR and the molecular energy gap of the acceptor. Increase in the intensity of these fields results in higher probability of optical transitions, thus engineering the optical properties of metallic nanostructures to achieve regions with high field intensity – referred to as hot spots – is desirable to increase the reaction rate [58]. In addition, as the near-fields are strongest at the NPs surface, reactant molecules should be within the evanescent volume of the plasmon fields for maximum efficiency [59], [60]. The rate of near-field induced transitions can be enhanced by the re-emitted photons from the radiative plasmon decay process [33]. Given the high multi-disciplinarity of the field, this process has also been referred to as plasmon-induced intramolecular excitation, direct intramolecular excitation, plasmon-pumped adsorbate excitation, adsorbate electronic excitation or plasmon-induced resonance energy transfer [59], [61].

#### 1.2.2 Hot carriers

The second non-thermal mechanism involves hot carriers. These are preferentially generated in regions where the electric near-field is strongest [62], [63], and during their short lifetime, they can be transiently transferred to accepting orbitals of nearby molecules. Provided that they have suitable energy and alignment with adsorbate orbitals, hot electrons (holes) can be transferred to the LUMO (HOMO) of adsorbed molecular species, driving reduction (oxidation) reactions. Generally, this transfer is reversible, resulting in the creation of a transient negative ion (TNI), which promotes the system along an electronically excited potential energy surface (*ψ*_E_ in Figure 1(b-ii)), thereby catalysing chemical reactions [10], [64], [65], [66], [67], [68]. Extending the lifetime of hot carriers within the acceptor orbitals of reactant molecules is a promising strategy to increase the probability of inducing a chemical reaction. Hot electron charge transfer can also initiate bond formation or drive redox reactions, in which case the ion or radical disassociates and the electron transfer is irreversible. Transfer can occur *indirectly* (Figure 1(b-iii), red arrows), where carriers are generated within the metal nanoparticles and then scattered into accessible molecular orbitals, provided they satisfy energy and momentum requirements [55], [69]. However, it is also possible for the energy levels of molecule–metal adsorbates to hybridize, allowing the *direct* excitation (Figure 1(b-iii), blue arrow) of hot carriers into these newly hybridized surface states [55], [69]. Hybridization affects the energy of the HOMO–LUMO, often leading to a reduced molecular gap (Figure 1(c-iii)) and opens a new energy transfer channel for hot carrier relaxation. Direct transfer, also referred to as chemical interface damping (CID) or plasmon resonant energy transfer [70], is a faster and more efficient process compared to the indirect transfer [33], [71], as it avoids energy losses due to scattering within the metal and during the injection process across the interface and is especially promising in terms of increased performance [33], [72], [73].

One of the promises of plasmonic photocatalysis is the possibility of achieving product selectivity by activating reaction pathways that are not attainable with conventional thermocatalysis [11], [73]. This can be achieved by providing energy via hot carrier transfer or near-fields to specific unoccupied adsorbate states, thereby promoting the system along an excited PES and facilitating targeted reaction pathways [15], [18], [73]. As shown in Figure 1(c), accessing excited states results in the modification of the reaction pathway corresponding to the production of different products. As near-fields and hot electrons energies depend on the physical properties of the plasmon nanostructures, the design and engineering of their optoelectronic properties is particularly important to achieve selectivity in multiproduct reactions such as CO_2_ reduction [74].

#### 1.2.3 Photothermal effect

Ultimately, hot carriers that not transferred to adsorbates will gradually lose their energy via scattering events releasing their energy as heat, thus leading to an increase in local temperature. This can enhance chemical transformation by increasing the population of reactants in vibrationally excited states (red arrows in Figure 1(c-ii)) and hence accelerating the reaction rate. As the reaction rate benefits from an increase in temperature, this energy transfer mechanism can be advantageous in many reactions for achieving higher product yields; however, as the photothermal effect drives the catalytic reaction along the ground state PES, it does not enable selectivity.

The temperature increase in a plasmonic system depends on the optoelectronic properties of the nanoparticles, their size, concentration and distribution of as well as the thermal properties of the surrounding medium and illumination conditions [1]. Taking into account these variables, the resultant temperature increase can be either confined in the vicinity of the plasmonic structures or – if collective effects are present – delocalized. In the latter case, the temperature profile is uniform throughout the whole nanoparticle assembly.

Given that the very fast timescales involved in the plasmon excitation and relaxation processes are similar to reaction time constants, disentangling thermal from non-thermal effects and understanding their relative contribution to plasmon reactivity has been object of a fruitful debate, as discussed in the next section [70], [75], [76], [77], [78].

### 1.3 Ongoing debate: photochemical versus photothermal

The primary subject of controversy is the elusive differentiation between thermal and non-thermal effects [19], [52], [54], [79]. One way to differentiate them would be to monitor local temperature increases, calculate the expected photothermal effect and deduce its impact on reaction rates using the well-known Arrhenius law. However, although it can seem a straightforward task, measuring plasmon-induced temperature increases on the surface of illuminated nanostructures is a challenging exercise because of the fast kinetics and small length scales involved, the complex light absorption and scattering pathways that affects mass and heat transport and large temperature gradients. When combined with factors relating to instrumental sensitivity, spatial resolution and uncertainty, it becomes apparent why this challenge proves demanding and has contributed to the ongoing debate.

**Figure 2:**
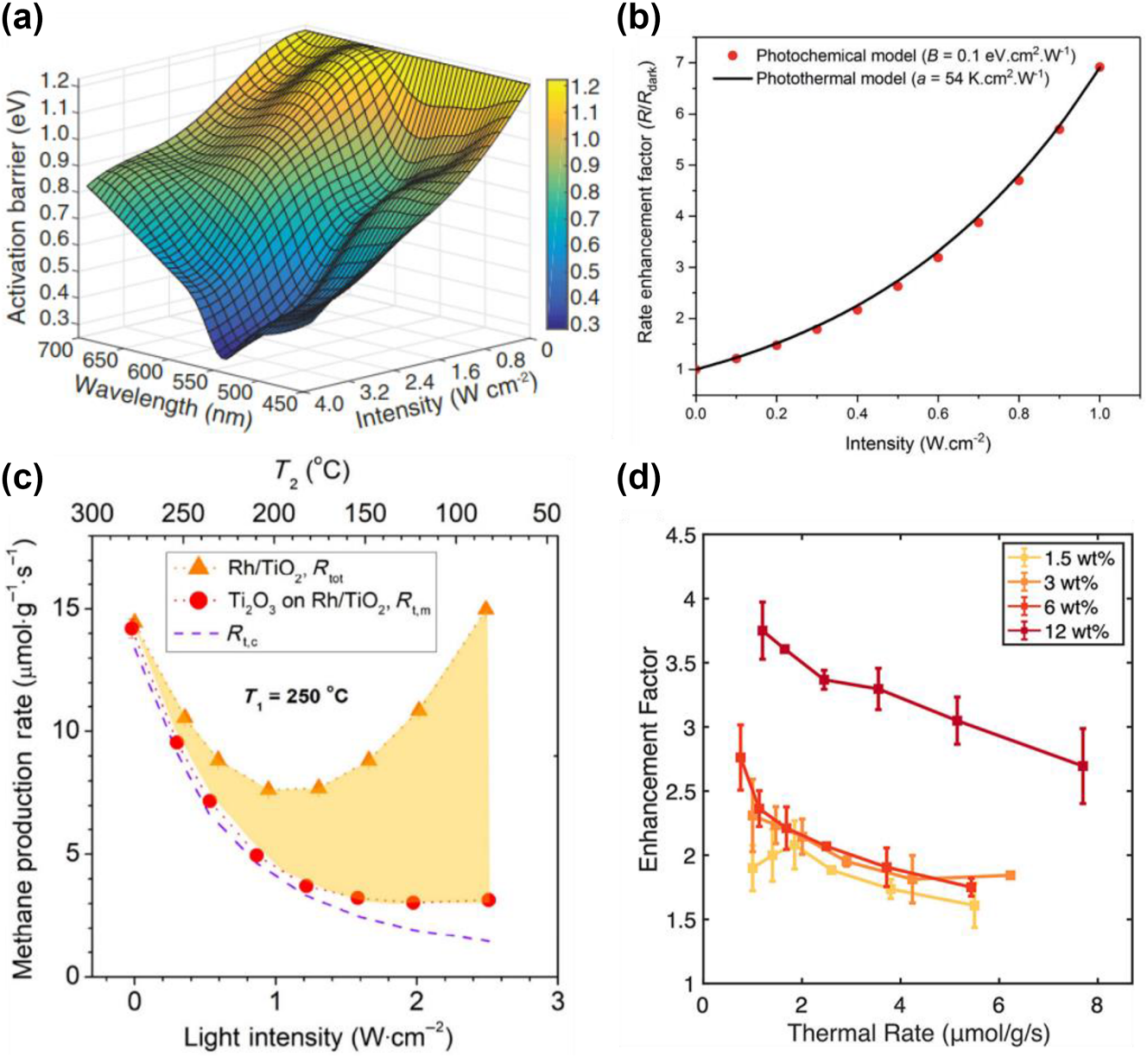
Photochemical versus photothermal debate. (a) Light-dependent activation energy, *E*_a_(*I*_inc, *λ*_) for the NH_3_ decomposition on Cu–Ru photocatalysts. (b) Reaction rate enhancement factor under plasmonic excitation fitted with a light-dependent activation energy and constant temperature (photochemical model, red dots) and with a light-dependent temperature but constant activation energy (photothermal model, black line). (c) Yield of CO_2_ transformed to methane on Rh–TiO_2_ photocatalysts, using direct (orange triangles) and indirect (red circles) illumination. The purple line represents the calculated thermal yield, while the shaded orange area represents the non-thermal contributions. (d) Enhancement factors (calculated by dividing the photothermal rates by respective thermal rates) as a function of the external heat supply for the CO oxidation on Ag/Al_2_O_3_ catalysts with different mass loadings. (a) Reproduced with permission from [64]. Copyright 2018, The American Association for the Advancement of Science. (b) Reproduced with permission from ref. [82]. Copyright 2020, Royal Society of Chemistry. (c) Reproduced with permission from [83]. Copyright 2019, Springer Nature. (d) Reproduced with permission from [84]. Copyright 2022, American Chemical Society.

The intricate interplay between thermal and non-thermal mechanisms has resulted in contrasting outcomes and divergent interpretations of data [80], [81]. Zhou et al. conducted a seminal study to quantify the contribution of hot carriers and thermal effects in enhancing ammonia (NH_3_) decomposition using Cu–Ru photocatalysts [64]. The authors measured the reaction rate under light illumination and in dark conditions with external heating and used a thermocouple to monitor the temperature of the sample surface. The results revealed a considerably higher reaction yield for the illuminated sample at comparable measured temperatures to those in the dark. By fitting the reaction rate with an Arrhenius law, the authors demonstrated that the activation energy is intensity and wavelength dependent under illumination (*E*_a_ (*I*_inc, *λ*_)) and reduces from 1.21 eV in the dark, to 0.35 eV at resonance, as shown in Figure 2(a). They concluded that thermal effects are not sufficient to explain the observed behaviour, and that the reaction is catalysed by plasmon-induced hot carriers. Nevertheless, the temperature evaluation within this approach was criticized: Dubi and colleagues suggested that the temperature measured by the thermocouple may not accurately capture the reaction conditions on the surface of the plasmonic photocatalysis. They went on to show that the reaction rates seen experimentally could be modelled using a purely thermal approach by fitting an Arrhenius equation with a constant activation energy (*E*_a_) and instead assuming an intensity dependent increase in the reaction temperature under illumination (∆*T* = *aI*_inc, *λ*_) (Figure 2(b)) [80]. This highlights that without an accurate measurement of the local reaction temperature, fitting experimental data to an Arrhenius equation does not provide conclusive proof that a chemical reaction is driven either by photochemical or photothermal effects [82].

Similar discrepancies [80], [85] are found in other works that have tried to discern between thermal and non-thermal effects for different reactions by means of standard macroscopic methodologies and surface-enhanced spectroscopies [86], [87], [88]. For instance, the plasmon-driven reduction of pNTP has been claimed to be driven both from non-thermal [89] and thermal effects [90]. Similarly, the N-demethylation reaction of methylene blue (MB) on plasmonic aggregates has been ascribed to both direct charge transfer of hot electrons [69] and enhanced plasmon near-fields, which pump energy into molecular adsorbates [57], [91].

#### 1.3.1 Experimental progress exploring the photothermal effect

To address the difficulty of accurately measuring the reaction temperature under illumination, Everitt and Liu developed a methodical experimental approach aimed at achieving identical thermal profiles with and without direct illumination [83]. They studied the plasmon-enhanced CO_2_ methanation on Rh/TiO_2_ catalysts in a system comprised of two thermocouples embedded in the catalyst reactor to accurately measure the temperature gradients (Figure 2(c)). They measured the reaction rate under standard direct illumination and under what they called ‘indirect’ illumination. The latter was achieved by using Ti_2_O_3_ as a photothermal heater: this material is inert (i.e. is not involved in the CO_2_ methanation reaction) and converts all the absorbed light into heat, allowing them to realize identical temperature gradients as in the case of the reactor without Ti_2_O_3_ (direct illumination), while suppressing the generation of near-fields and hot electrons. The authors found that the combination of thermal and non-thermal effects can synergistically enhance the reaction rates of CO_2_ methanation on Rh/TiO_2_ catalysts, and that non-thermal contributions are significant. Interestingly, when adopting a similar approach for the NH_3_ decomposition on ruthenium catalysis, they found that the reaction was mainly driven by thermal effects [92].

Another rigorous approach to distinguish between these effects was recently proposed by Elias et al. [84]. By developing an annular quartz tube photoreactor with a controllable rate of mass transport and with thermocouples embedded in the catalyst bed, they were able to accurately measure the macroscopic equilibrium temperature and CO oxidation rate on Ag/Al_2_O_3_ catalysts *in situ*. Investigation of the reaction kinetics under external heating and visible light illumination revealed that the rise in equilibrium temperature by itself could not explain the enhanced reaction rate observed during illumination (Figure 2(d)). This remains true even at elevated catalyst loading with larger metal nanocluster sizes (>6 wt%), where photothermal heating is known to be greater and particles are shown to have collective effects [17], [93]. Based on the results, the authors proposed that highly localized effects play a critical role in driving the plasmon-enhanced chemical reactions, including strong near-fields and electronic excitation of reactants from highly localized photothermal heating.

Ultimately, to disentangle these effects, care should be taken while designing the reaction conditions and accurate methodologies should be employed to quantify the co-existence of thermal and non-thermal effects. These can range from simple experimental procedures [53], [94], to more elaborate computations and methodologies [95], [96], [97], [98].

Commonly used techniques to measure the temperature increase in plasmonic systems can be categorized into surface-averaged and single-particle methodologies [99]. The former are beneficial when collective effects are present and the temperature profile is homogenized and include the use of thermocouples, thermoreflectance measurements and thermal cameras. Thermocouples and thermoreflectance measurements translate temperature changes into electric signals or reflectance differences, offering a spatial resolution of approximately 1 µm. In contrast, thermal cameras convert infrared energy into an optical image with a spatial resolution of around 100 µm. Due to their simplicity and minimal impact on the system, these are often relied upon for temperature measurements during catalytic gas-phase reactions [19], [48], [52], [64], [100], [101]. The latter are often used when higher resolution is needed, for instance during the mechanistic investigation of plasmon-mediated chemical reactions via ultra-fast spectroscopy [89] or surface-enhanced Raman scattering [69]. These rely on anti-Stokes thermometry [102], [103], [104] and spectrally measure the anti-Stokes photoluminescence of metallic nanoparticles or probe molecules adsorbed on the surface of plasmonic elements to evaluate the temperature increases of individual nanoparticles *in situ*. Other techniques with high spatial resolution and single-particle capability are scanning probe microscopies including atomic force and scanning thermal microscopy, where the temperature increase is measured through conductivity changes or using the tip as a thermocouple [105], [106].

Despite the experimental difficulties in distinguishing between non-thermal and thermal effects, careful experimentation has repeatedly shown that the mechanisms responsible for plasmon-assisted catalysis depend on the reaction studied, and most importantly, that the different non-thermal and thermal effects can cooperate synergistically to drive or enhance the catalytic process [9], [51], [107].

### 1.4 Near-fields or hot electrons?

**Figure 3:**
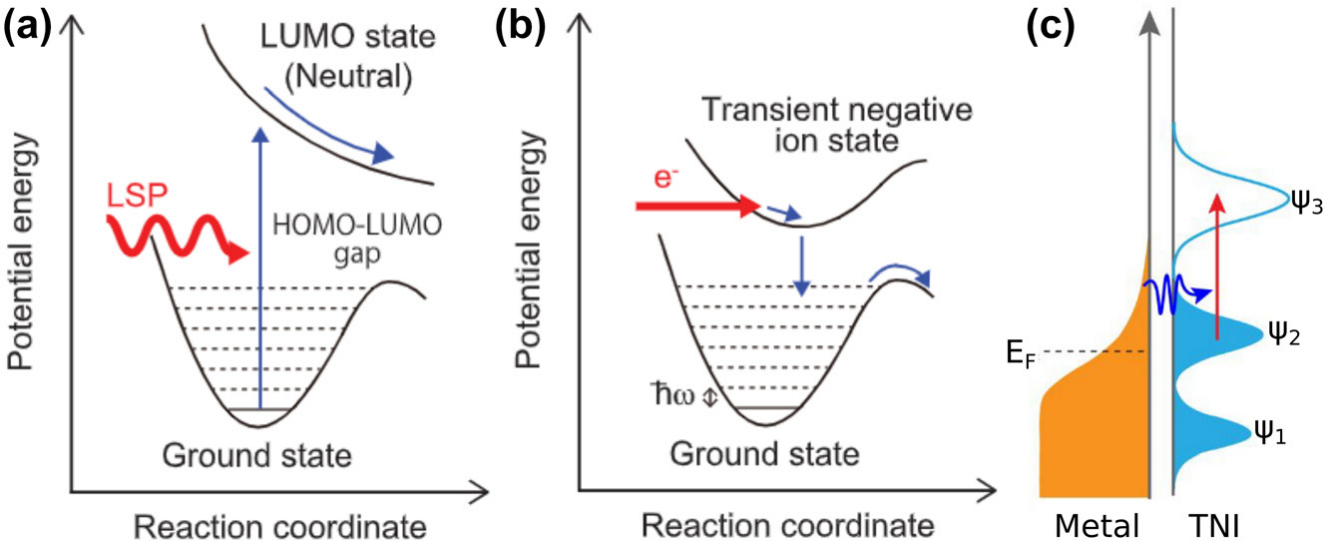
Near-fields and hot electrons energy transfer. Subtle difference in reaction pathways driven by direct intramolecular excitation (a) and hot electron transfer to induce a TNI (b). (c) Proposed energy transfer mechanisms involving near-field enhanced excitation of TNIs prepared through charge transfer processes. *ψ*_i_ represents the electronic state of the adsorbate. (a–b) Adapted with permission from [96]. Copyright 2018, the American Association for the Advancement of Science (c) Adapted with permission from [110]. Copyright 2020, American Chemical Society.

As previously discussed, non-thermal energy transfer between adsorbed molecules and plasmonic structures can be mediated by optical near-fields and hot carriers and can promote the system to an excited potential energy surface (PES). Both processes benefit from interaction – or hybridization – between metal states and frontier molecular orbitals; however, the strength of hybridization can favour one mechanism or the other. In a recent work, Kazuma et al. employed scanning tunnelling microscopy (STM) to monitor the dissociation reaction of dimethyl disulfide ((CH_3_S)_2_) on Ag and Cu surfaces in real-space and real-time [96]. The authors performed measurements under different illumination and bias conditions and found that the reaction yield strongly correlates with the near-field intensity of the optically excited LSPR. They demonstrated that this reaction was driven by direct intramolecular excitation by combining wavelength-dependent reaction yield experiments with finite-difference time-domain simulations to compute the near-field distribution and density functional theory (DFT) to investigate the electronic structure and molecular orbitals of (CH_3_S)_2_ adsorbed on metal surfaces. In this case, the strong plasmonic near-fields generated at the nanogaps between the STM tip and the metal surface drive charge excitation from the molecular HOMO–LUMO, as opposed to the transfer of hot electrons from the metal to the molecule. This energy transfer was accessible despite the fact that light with energy below that of the HOMO–LUMO of isolated (CH_3_S)_2_ because (CH_3_S)_2_ weakly hybridizes with the metal substrates, thus reducing its optical energy gap [108] and promoting the reaction through the pathway in Figure 3(a).

In contrast, the dissociation of strongly hybridized small molecules – such as H_2_ and O_2_ [86], [109] – on plasmonic nanoparticles is attributed to hot-electron transfer. Even though the HOMO–LUMO gap of these molecules is accessible only by UV light, these dissociation reactions have been observed to occur under visible-light irradiation on plasmonic catalysts. Density functional theory computations were used to investigate the role and feasibility of hot electron transfer from the metal NPs to the antibonding orbital of these adsorbed molecules and supported the experimental results. These studies demonstrated that high-energy electrons transferred to the antibonding orbitals of H_2_ and O_2_, generating TNIs (H_2_^δ−^ and O_2_^δ−^), exciting the system to a higher PES and driving the dissociation reaction along the pathway in Figure 3(b).

Recently, an interesting point of view has been proposed in the discussion on plasmon reactions driven by non-thermal effects [110]. Building on evidence from the N-demethylation reaction of methylene blue [57] and knowledge from gas phase dissociation reaction of CO_2_ [111], Habteyes suggested that near-fields play a more significant role in plasmon-driven photochemical reactions than hot electrons [110]. He takes issue with the current implication in the literature that the TNIs formed by hot carrier transfer undergo an automatic and spontaneous chemical transformation and thus that the plasmon action mechanism is often ascribed to be driven by hot carriers. In contrast, this work proposes that most, if not all, plasmon-mediated reaction are driven by direct excitation of adsorbates and adsorbate–metal complexes by near-fields (Figure 3(c)). He argues that the role of charge transfer is limited to preparing the TNIs: electron transfer results in anionic complexes with the form of *S∙[Molecule-L*_*n*_ *]* ^*−*^ (where *S* is the metal surface, *L* can be a ligand or an environmental molecule and *n* is an integer), which are stabilized through surface–molecule-environment interactions, after which the reaction proceeds via near-field energy transfer. These newly formed anionic complexes act as intermediate species and could explain the apparent reduction of activation barriers [110]. This mechanism assumes hybridization between the metal NP and reactant species and implies some degree of cooperation between multiple mechanisms.

This was recently confirmed by using Au dimers with varying interparticle distances from 5 nm to 30 nm to monitor the dehalogenation reaction of 4-iodothiophenol (4-ITP) [112]. By using *in situ* SERS spectroscopy, the authors found that the gap size significantly affects the reaction kinetic. Reducing the separation of the Au dimers from 30 nm to 10 nm resulted in an approximately fourfold increase in the reaction rate. The authors demonstrated that the reaction is predominantly governed by the hot-electrons generated at the gaps, and that stronger near-fields are crucial for efficient hot carrier generation. Similarly, the synergy between strongly confined near-fields and enhanced hot carriers generation has been demonstrated in bimetallic plasmon photocatalysts for the production of hydrogen [113]. This work showed that Au–Pd core-satellite architectures achieve better H2 generation performance compared to core–shell systems, due to the excitation of highly localized and asymmetric near-fields in the gap of core-satellites structures [113].

As the near-fields have a significant impact over the generation of hot carriers through increased photon absorption at the metal surface, balancing the near-fields enhancement and absorption of the plasmonic system is crucial to develop effective photo-activated catalysts [114], [115].

In summary, there is an increasing body of literature that agrees on the synergistic role of non-thermal effects in driving or enhancing chemical reactions. However, their relative weighting is still unclear and likely depends on the system. Detailed theoretical methodologies could help answering this question.

## 2 Recent progress in atomistic modelling of chemical reactions

### 2.1 Time-dependent density functional theory

To complement the experimental evidence of plasmon-assisted photocatalysis and to better understand the mechanisms underlying plasmon photocatalysis, considerable effort has been devoted to the study and prediction of excited-state processes. While the optical response of plasmonic structures is accessible with classical electromagnetic simulations [17], [27], [58], investigation of the dynamics of excited plasmon states requires complex quantum approaches. In recent years, significant effort has been devoted in developing theoretical frameworks for the hot carrier generation in plasmonic structures.

Seminal works have framed the current understanding of hot electron generation using different levels of approximation [116], [117], [118], [119]. Govorov et al. developed a simple theoretical framework based on an electron-gas model for various geometries to evaluate the initial energy distribution of hot-carrier generated by plasmon decay [62], [119]. Manjavacas et al. used a jellium approximation to study the plasmon-induced hot carrier process and extended the results to spherical Ag nanoparticles and nanoshells [118]. Atwater’s group have used an *ab initio* methodology accounting for a detailed electron structure to improve the electronic description of the system [116], [117], while Bernardi et al. [120] combined DFT and electron–phonon calculations to study the energy distribution and scattering processes of the hot carriers generated by SPP in flat surfaces of Au and Ag. All these works contributed to the current knowledge on hot carriers and share similar trends in energy distribution and their dependency on material, band structure and geometry.

**Figure 4:**
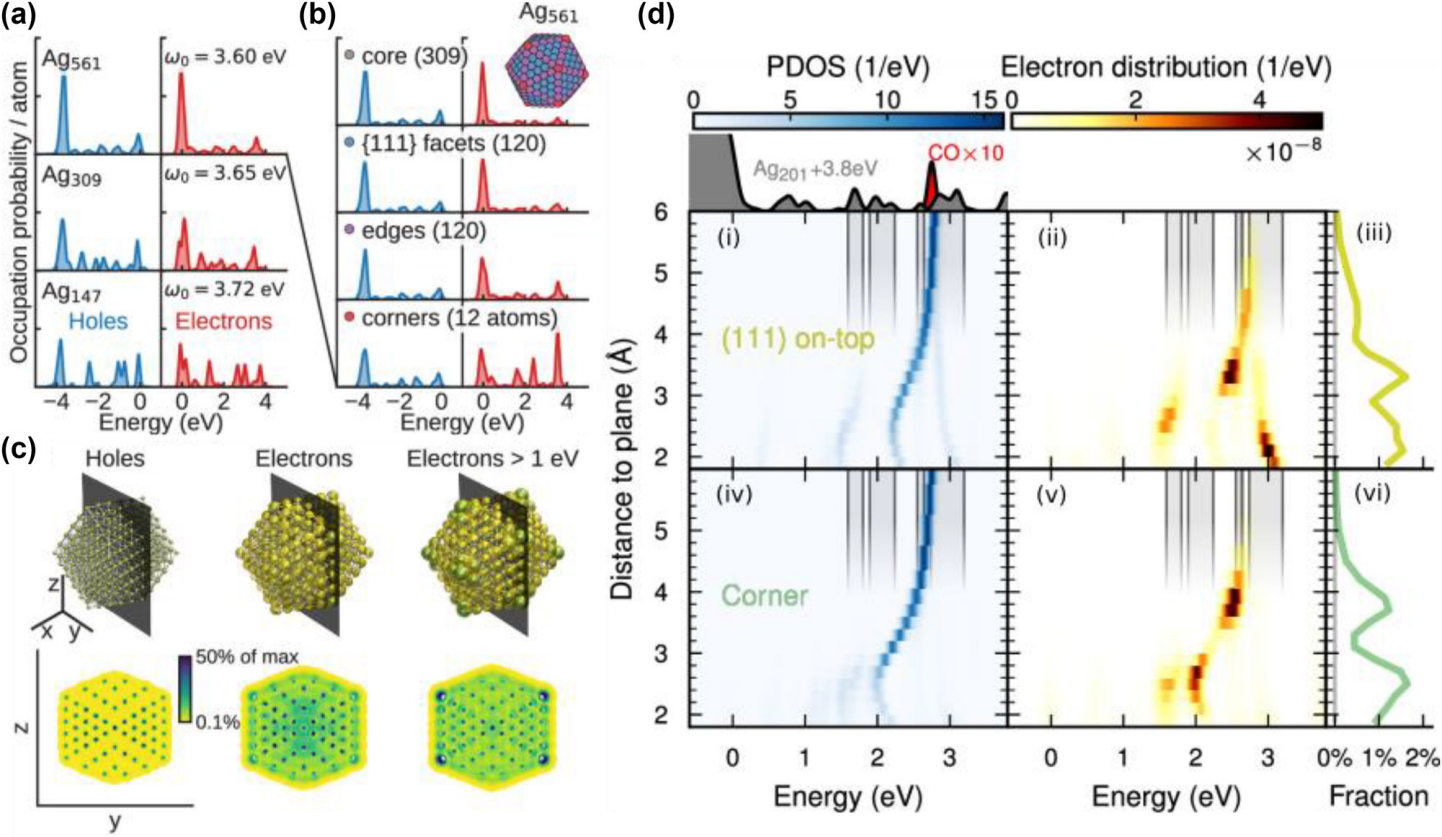
Hot carriers distribution and transfer from TDDFT. (a) Hot carrier energy distribution for Ag clusters with different sizes. (b) Probability of hot carrier generation at different atomic sites normalized for the number of atoms for an Ag_561_ cluster. (c) Spatial distribution of hot holes, hot electrons and hot electrons with energy >1eV for a Ag_561_ cluster. (d) Molecular PDOS, hot electron distribution and transfer probability as a function of the distance for Au201 + CO on a (111) on-top site (i-iii) and for the same for CO on a corner site (iv-vi). (a–c) Reproduced with permission from ref [41]. Copyright 2020, American Chemical Society. (d) Reproduced with permission from [121]. Copyright 2022, American Chemical Society.

These frameworks prompted further advances in atomic-scale modelling of plasmonic hot carrier generation and transfer to molecules or semiconductors, a subject of significant importance in photocatalysis. In series of recent publications, Rossi et al. have developed a fully atomistic methodology to study the plasmon dynamics and electronic excitations of discrete plasmonic systems using a real-time time-dependent DFT (rtTDDFT) approach [41], [121], [122], [123]. The authors have thoroughly analysed the energy and spatial distribution of hot carriers in Ag clusters and showed a pronounced dependence on the size and local structure (Figure 4(a–c)). Upon excitation with a laser pulse at the LSPR frequency, they demonstrated that, as the size of the cluster increases from ∼150 atoms to over 500, the hot carrier distributions are increasingly controlled by interband d-electron transitions and eventually converge to the distributions obtained for flat surfaces [116], [120]. In addition, smaller clusters show a higher population of high-energy carriers, arising from the stronger contributions of sp-states. Interestingly, they found that the spatial distribution of holes is relatively uniform within the cluster with negligible differences between bulk and surface states, while the electrons are more delocalized and reside to a larger extent in the surface region (Figure 4(c)). In particular, lower-coordinated sites (edges and corners) showed larger occupational probability for energetic hot electrons compared to bulk states.

These results support the concept of lower-coordinated sites being more catalytically active in heterogeneous catalysis [124], [125] and justify the quest for morphologies with high curvature [58], as these structures support stronger near-field and superior hot electron generation. This rtTDDFT methodology has been used to investigate the direct charge transfer from plasmonic nanoparticles to adsorbed CO molecules and bulk CdSe semiconductors [72], [126], demonstrating plasmon direct hot electron transfer with efficiencies up to 2 % for the CO molecule and 23 % for CdSe. The significant difference in efficiency is due to the higher density of states (DOS) of the CdSe compared to CO.

Interestingly, while it was previously thought that molecules had to be chemisorbed to the metal surface to enable hot carrier transfer [68], a recent rtTDDFT study [121] from the same group has shown that CO molecules can form hybridized energy states with metal nanoclusters at distances up to 6 Å, allowing direct electron transfer (Figure 4(d)). They showed that hot electron transfer depends non-monotonically on the NP–molecule distance and can be effective at long distances, even before a chemical bond can form. Fine tuning the cluster morphology to achieve spectral overlap between optical excitation the energy and the LSPR can lead to direct electron transfer efficiencies up to 8.9 % for a Ag nanocluster and a CO molecule at 2.7 Å from its surface. They further demonstrated that the distribution of hot electrons directly generated on the molecule mirrors the projected density of states (PDOS) for the hybridized metal cluster-molecule system at ground state. The authors point out that the PDOS is a sufficient indicator for a qualitative prediction of the energy distribution of photogenerated hot carriers, suggesting that ground state calculations can be used as a first approximation to quickly screen potential candidates for photocatalysis.

Similar results on the importance of hybridization for electron transfer were found by Le et al. [127] during the investigation of the plasmon-induced CO_2_ conversion on Al@Cu_2_O by ground- and excited-state DFT, using the delta self-consistent field (ΔSCF) approximation. This approach allows the calculation of the electronic structure and the energy of excited states by iteratively adjusting the electron density and wavefunction, allowing for predictions of excitation energies and dynamics in molecular systems. They showed that hybridization between CO_2_ and Cu_2_O creates new CO_2_ antibonding states at energies below 2.2 eV, thus accessible by direct plasmonic-excited hot electrons generated by visible light excitation.

The relative contribution of direct and indirect charge transfer process has been recently investigated by Zhang et al. [128] by real-space TDDFT coupled with non-adiabatic Ehrenfest molecular dynamics for CO_2_ reduction on Ag_20_ and Ag_147_ icosahedral clusters. By analysing the time-evolution of Kohn–Sham states, they showed an interplay between direct and indirect electron transfer to unoccupied CO_2_ levels, which can be tuned by using different laser powers. At lower laser intensity (<250 mJ cm^−2^), indirect and direct charge transfer mechanisms cooperatively promote CO_2_ reduction with a relative contribution of about 60 % and 40 %, respectively, while at high laser intensities (>350 mJ cm^−2^), the direct transfer is highly favoured with a contribution close to 100 %. Furthermore, they showed that larger Ag_147_ clusters are faster in breaking the C=O bond of adsorbed CO_2_ compared to Ag_20_ clusters (40 fs vs 80 fs); this was attributed to an intensified electronic density of states near *E*_F_ with the increase of the cluster size, which give rise to stronger plasmon field, thereby accelerating the reaction [129].

### 2.2 Other computational approaches

Although DFT calculations are considered the workhorse of *ab initio* quantum mechanics methodologies, their results are only as good as the level of approximation used. As such, the results from these studies can often only provide guidelines for understanding the processes investigated [130], [131]. This is because of limitations in properly describing the intricate many-body effects, which are approximated by the electron exchange–correlation (XC) functionals.

In CO_2_ electroreduction on transition metals, it is widely accepted that the final product distribution depends on the binding energy of CO on the catalyst surface [132], [133]. Formation of CO is considered the rate-limiting step of the CO_2_ reduction, and the binding energy of this key intermediate is often computed by DFT methods. However, inaccuracies inherent in XC functionals (i.e. the self-interaction error) leads to an erroneous estimation of the CO 2π* orbital energy, which in turn leads to the incorrect predictions of the CO adsorption site and free energy [134]. Instead, more accurate methodologies based on the embedded correlated wavefunction (ECW) theory [135] can be used to account for electron–electron correlation effects and can more accurately describe hybridization and charge transfer processes. While this approach has been used to investigate plasmon-mediated chemical reactions including NH_3_ decomposition [136], [137] dissociation of small molecules [86], [138] and C–H and C–F bond activation [97], [139], there have been no reports of ECW applied to plasmon photoreduction of CO_2_.

This theory has been used to revisit the understanding of electrochemical CO_2_ reduction on copper [134], [140]. By employing the EWC theory, Zhao and colleagues found that the first step in CO reduction on Cu(111) involves *COH instead of the previously thought *CHO, with *COH formation kinetically preferred over *CHO formation by 0.84 eV. This could lead to the reaction happening on a different potential energy surface, possibly leading a different product distribution.

Unfortunately, a major limitation of this methodology is that it is computationally demanding, which does not allow for a facile scale-up. To mitigate this, a recent report by Chen and colleagues [141] demonstrated highly accurate computational CO_2_ reduction on copper surfaces, benchmarked against several experimental, theoretical and analytical results. This was achieved using a hybrid DFT scheme that combines the doubly hybrid XYG3 XC functional with the periodic generalized gradient approximation and has the advantage of achieving high precision in describing metal–molecules interaction. As this XC builds on top of existing DFT methodologies, it has the potential of being easily implemented and scaled-up for larger systems and could lead to a rapid acceleration of computational chemistry for finite and extended systems in heterogeneous catalysis [142].

Extending these findings to the photoreduction of CO_2_ and other reactions afforded by plasmonic materials is of pivotal importance in progressing the field of hot carrier science, potentially enabling a predictive comprehension of excited state metal surface reactions.

## 3 Recent progress in experimental CO_2_ reduction

Solar-driven conversion of CO_2_ and H_2_O into valuable chemical fuels is a very promising approach to address current energy and environmental challenges. Using hydrogen produced by water splitting and CO_2_ directly captured from the air enables the opportunity to synthesize virtually any valuable hydrocarbon or alcohol.

The challenge in achieving commercially viable production of such valuable fuels is realizing high yields of the desired product in a scalable system and is a critical object of research. In the following sections, we will discuss the most recent advancements on the use of plasmon catalysts for CO_2_ reduction. We will organize the experimental finding into two categories: *in situ* measurements, which are primarily used to investigate the reaction mechanisms, and ensemble measurements, which aim to demonstrate optimal performance. In this context, ensemble studies refer to research that adopts macroscopic analytical tools like gas chromatography (GC) and mass spectroscopy (MS) to quantify the product distribution. In the quest to understand the plasmon action mechanisms and engineer efficient plasmonic photocatalysis, many works have used a combination of *in situ* and ensemble measurements. Consequently, this section will not provide an exhaustive review of all research on plasmon-enhanced photocatalysis, but rather it aims to offer an informative and succinct summary of experimental methodologies for probing reaction pathways and mechanisms, as well as to showcase the significant contributions made by pioneering and cutting-edge research in this field. For a comprehensive introduction to experimental characterization techniques for plasmon-assisted chemistry, we refer the readers to the excellent Reviews [99], [143], [144].

### 3.1 *In situ* measurements

*In situ* techniques enable detailed insights into the structure–activity relationship of catalysts and are commonly used to investigate reaction pathways, intermediates and mechanisms. This is crucial for designing efficient, selective and stable catalytic systems.

#### 3.1.1 Vibrational spectroscopies

Vibrational spectroscopies – including Raman and infrared (IR) spectroscopies – are amongst the most widely used *in situ* techniques and are used to identify structural information of molecules adsorbed on surfaces. They generate spectra that capture the characteristic vibrations of molecules (fingerprints), enabling non-invasive monitoring of the dynamics of chemical reactions with sub-second resolution. Owing to their high sensitivity and specificity, these techniques are useful to obtain information on possible reaction pathways. Raman spectroscopy is based on inelastic scattering of incident monochromatic light by a molecule and relies on the change of its polarizability. The energy of the scattered light is shifted by an amount corresponding to the vibrational energy of the molecules and this shift provides detailed information about the chemical bonds, molecular structure and composition of the material. The IR and Raman cross sections represent the likelihood of the respective phenomena and are extremely small [145] (10^−20^ and 10^−30^ cm^−2^, respectively) for most molecules; however, they can be greatly enhanced by using resonant metallic structures.

**Figure 5:**
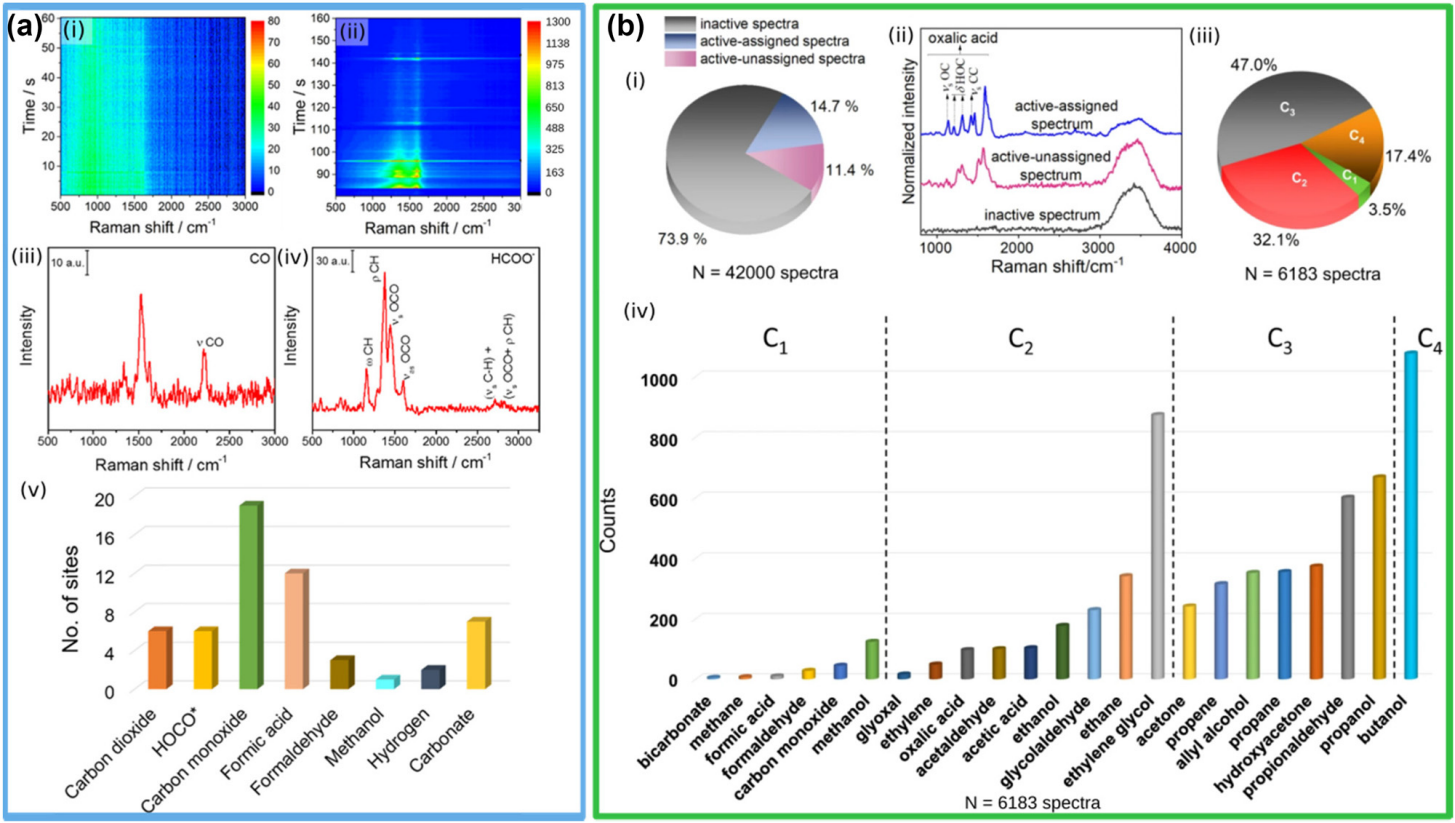
*In situ* SERS photoreduction of CO_2_ on Ag clusters. (a-i, ii) Temporal maps showing continuously acquired *in situ* SERS spectra of air in CO_2_, respectively. (a-iii, iv) Representative SERS spectra that capture some (CO and HCOO-) of the many products observed during the photocatalysis. (a, v) Amount of observed specie in CO_2_ photoreduction. (a) Reproduced with permission from [146]. Copyright 2018, American Chemical Society. (b) Reproduced with permission from [147]. Copyright 2021, Springer Nature.

The strong and highly localized near fields generated at the surface of metallic nanoparticles are routinely used to amplify the vibrational Raman signal of molecules, in a technique known as surface-Raman enhanced spectroscopy (SERS) [32], [37]. This technique has been used by Kumari et al. to investigate the dynamics of CO_2_ reduction and products on Ag NPs under visible light illumination [146]. The authors have used a microfluidic flow cell and performed gas-phase SERS measurements with a 514.5 nm (∼2.4 eV) continuous-wave (CW) laser, matching the LSPR of Ag aggregates, in air and CO_2_ atmospheres (Figure 5(a, i-ii)). When exposed to air with a 30 % humidity, they observed a broad featureless scattering spectrum that remains steady with the time. Conversely, upon introduction of CO_2_, a stochastic appearance and disappearance of distinct vibrational fingerprints was observed (Figure 5(a iii–iv)). DFT calculations were used to assign the observed vibrational modes to different chemical species. The product distribution of such species is shown in Figure 5(a-v), indicating the presence of multi-electron reduction species such carbon monoxide (CO) and formic acid (HCOOH), as well as simple alcohols. Importantly, this work identified the very first step of plasmonic CO_2_ reduction on Ag, by observing the key surface intermediate hydrocarboxyl (HOCO*) originating from the transfer of 1e^−^ from the Ag nanoparticle to CO_2_. Furthermore, they showed that CO_2_ is weakly bound to the Ag nanoparticles, at a distance of ∼3.4 Å away from the surface of the Ag nanoparticles. This implies that even over relatively long distances, electron transfer between the metal and the CO_2_ molecules occurs, in agreement with the theoretical calculations discussed in Section 3.

The same group have used *in situ* SERS on similar Ag NPs to study the photoreduction of CO_2_ in the liquid phase, using water as a reaction medium under 514.5 nm CW illumination, in an attempt to extend the product distribution of plasmonic catalysis [147]. They rigorously analysed 42,000 SERS spectra and were able to detect a wide range of products and intermediates, including multi-carbon (C_1_–C_4_) species. Out of all the collected spectra, just ∼26 % showed vibrational fingerprints different from the control experiments, and ∼15 % were identified (Figure 5(b, i-ii)). The assignment of the different peaks in the SERS spectra was thoroughly validated through DFT computations and isotope measurements using ^13^C. The results of these measurements are striking: although Ag NPs yield mostly CO as a product, out of the active spectra, 96.5 % of them show multi-carbon products (Figure 5(b, iii)), leading the authors to infer that multi-electron transfer and C–C coupling is favourable on plasmonic-excited NP surfaces. The relative abundance of the detected species categorized by the number of their constituent carbon atoms, as shown in Figure 5(d, iv), demonstrates the presence of a wide range of highly valuable species, like ethanol, ethylene, acetone, propanol and butanol.

This study marked the first identification of C_3_ and C_4_ compounds on Ag-catalysed reactions and holds significant implications for plasmonic CO_2_ photoreduction; however, it is important to note that the abundance depicted in Figure 5(b, iv) is just an occurrence count and it does not provide a quantitative product distribution. Additionally, the authors used high power density laser (10^8^ Wm^−2^) to investigate the elementary steps and intermediate species of CO_2_ reduction in an aqueous environment: this facilitated multi-photon excitation and carrier re-excitation processes, which would result in the generation of highly energetic electron–hole pairs comparable to those generated by UV light. Furthermore, the detected species are only present in trace amounts and do not necessarily survive or contribute to the final product profile. However, showcasing a rich catalogue of C_2+_ species, this study has given a new perspective of the field of plasmonic photocatalysis with respect to the possibility of converting CO_2_ into valuable C_2+_ products.

*In situ* infrared spectroscopies are complementary techniques to SERS and include Fourier-transform infrared spectroscopy (FTIR), diffuse reflectance infrared Fourier transform spectroscopy (DRIFTS) and surface-enhanced infrared absorption spectroscopy (SEIRAS). Contrary to SERS, these rely on absorption of infrared light and depend on the change in the dipole moment of a molecule [148]. Whether or not a vibrational mode is active or inactive for Raman or IR techniques depends on the symmetry of the molecules. As IR absorption is particularly sensitive to heteronuclear molecules and polar bonds this technique is more suitable for analysing liquid-phase catalytic reactions, thanks to its high sensitivity to water and OH groups. These technique allow for in-operando conditions and have been widely adopted to investigate the kinetics of electrochemical reduction of CO_2_ [149], [150], [151], providing important information on surface adsorbates and intermediates, and are recently being translated to the photocatalysis field [8], [152], [153], [154], [155], [156].

**Figure 6:**
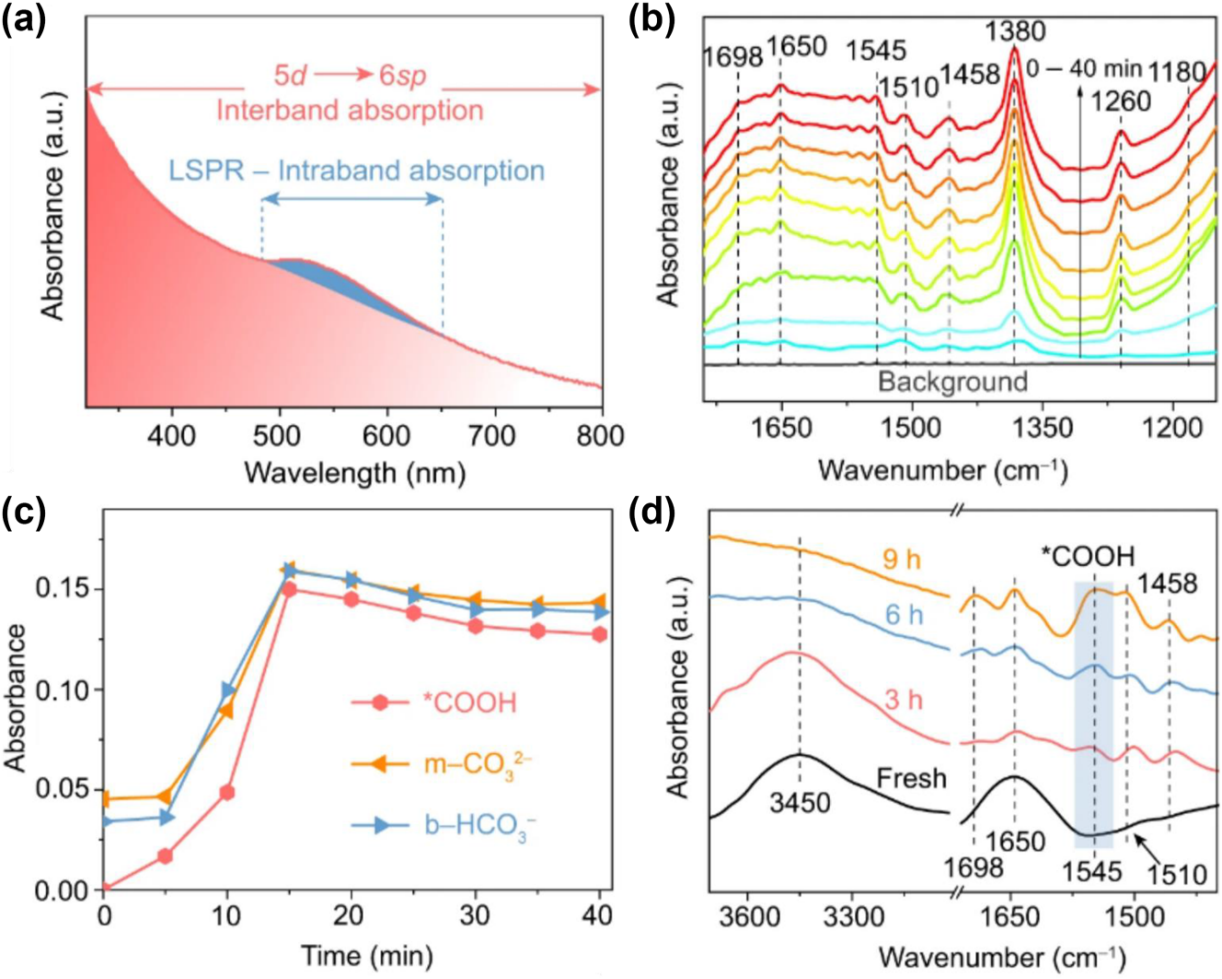
*In-situ* monitoring of reaction intermediates with vibrational spectroscopy. (a) Relative contribution of interband and intraband transition in quantum-sized Au NPs. (b) *In situ* FTIR spectra of the photocatalytic CO_2_ reduction process occurring with H_2_O on Au. (c) Time-evolution of the absorbance of key reaction intermediates. (d) FTIR time-steps of the photocatalytic reaction of CO_2_, showing strongly adsorbed *COOH after 9 h of reaction. Reproduced with permission from [154]. Copyright 2022, Springer Nature.

A recent work by Shangguan et al. [154] reported that Au NPs with a plasmon response at ∼540 nm (Figure 6(a)) efficiently reduce CO_2_ to CO in presence of H_2_O, showing more than a twofold rate increase in H_2_O compared to H_2_. This is a surprising result as generally the photocatalytic conversion of CO_2_ on metal NP in water requires reducing agents [157], [158], and it is hindered by the competing hydrogen evolution reaction. To gain molecular insights, the authors performed *in situ* FTIR measurements (Figure 6(b–d)).

A broad peak centred at ∼1650 cm^−1^ and attributed to H_2_O appears with the increase of the reaction time, suggesting that H_2_O was adsorbed on the Au NPs surfaces. Simultaneously, CO_2_ was activated by the plasmonic NPs, as evidenced by the CO_2_^−^ peak at 1698 cm^−1^. When two or more of these species are in close proximity, they can interact to form monodentate carbonate (m-CO_3_^2−^ identifiable from the strong peak at 1380 cm^−1^ and at 1510 cm^−1^) and bidentate bicarbonate (peak at 1458 cm^−1^). As the reaction progresses, further signatures of adsorbed *COOH appear with time (peaks at 1545 and 1180 cm^−1^), as shown in Figure 6(c). This peak is retained for up to 9 h, indicating that *COOH is strongly adsorbed on the active sites, thereby contributing to the suppression of the HER. The authors supported these results with DFT computations and proved that the activity is induced by surface Au–O species formed from H_2_O decomposition, which simultaneously optimize the rate-determining steps in the CO_2_ reduction and H_2_O oxidation reactions, lowers the energy barriers for the *CO desorption and *OOH formation and facilitates CO and O_2_ production.

Similar results were found while assessing the photocatalytic performance of plasmonic Au/CdS, Cu/Cu_2_O octahedrons and Ag, Cu/β-Ga_2_O_3_ [159], [160], [161]. *In situ* DRIFT measurements were employed to assess the intermediate species formed during the CO_2_ conversion, which led to selective CO production with yields of a few tens of μmol g^−1^ h^−1^. These studies identified adsorbed bicarbonate (HCO_3_^−^) and COO^−^ in the wavenumber range of 1350–1520 cm^−1^ and 1650 cm^−1^, suggesting that these are important intermediates in the CO_2_ reduction which can readily accept protons and electrons to form CO.

#### 3.1.2 Electron spectroscopies

As chemical reactions happen at the interfaces, *in situ* monitoring of the binding energy between reactants and surface atoms offers valuable insights into the chemical state changes occurring during a reaction and driven by electron exchange, helping understanding reaction mechanisms and kinetics. Near-ambient pressure-X-ray photoemission spectroscopy (NAP-XPS) is a powerful technique that can be used to monitor the surface chemistry with unprecedent resolution while the reaction is occurring [162], [163]. A recent example is the assessment of the role of plasmon excitation in Au@CuPd core–shell composites for the reduction of CO_2_ to CH_4_ in water environments [164]. Hu and colleagues achieved ∼100 % selectivity toward CH_4_ under 400 mW cm^−2^ full-spectrum light illumination using Au nanorods as light-harvesters combined with catalytically active CuPd alloys shells.

**Figure 7:**
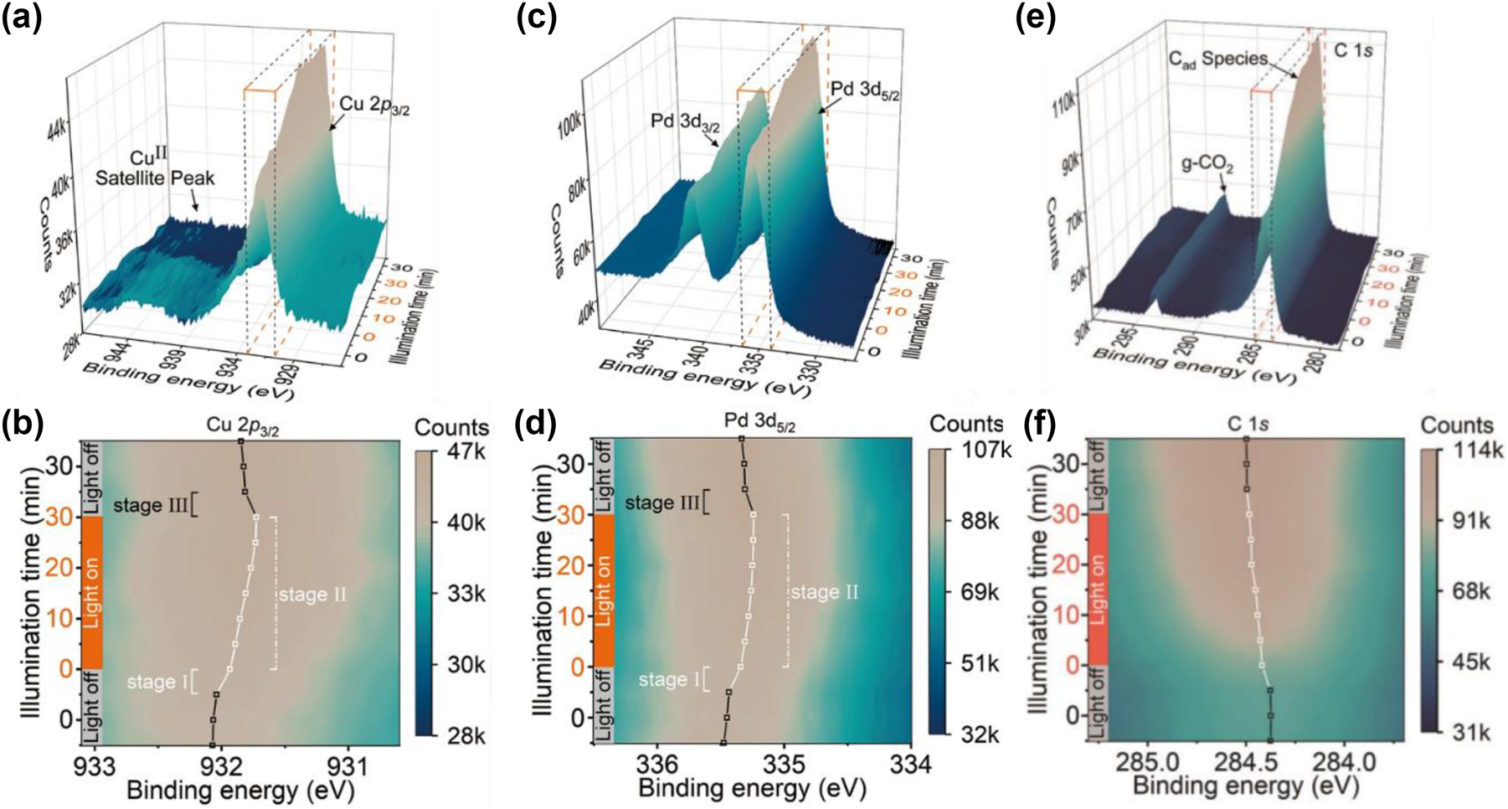
*In-situ* monitoring of surface chemistry during CO_2_ methanation. (a, b) In situ NAP-XPS spectra of Cu 2p3/2, (c, d) Pd 3d5/2 and (e, f) C 1s. Panels b, d and f show a top-view contour plot of the highlighted regions in (a, c and e), respectively. Reproduced with permission from [164]. Copyright 2023, Springer Nature.

Interestingly, upon monochromatic illumination at 800 nm (∼1.55 eV), the authors measured a stable CH_4_ production rate over 10 cycles with an apparent quantum efficiency of 0.38 %. The results of this research represent the state-of-the-art of CO_2_ reduction under NIR illumination. To gain insights into this low-energy photon utilization, the authors have used *in situ* NAP-XPS. The results of these measurements are shown in Figure 7. Upon light irradiation, the binding energy of the Cu 2p3/2 and Pd 3d5/2 peaks decreased (Figure 7(a–d)), indicating the reduction of Cu and Pd species. This was attributed to accumulation of hot electrons generated by Au LSPR relaxation above the Fermi level (*E*_F_). Simultaneously, the peaks attributed to adsorbed carbon and gaseous CO_2_ (Figure 7(e and f)) increase in intensity and present a positive shift, indicating adsorption of CO_2_ molecules and their conversion process to hydrocarbons. Combined with DFT simulations, the authors concluded that hot electrons accumulated above the *E*_F_ because of the presence of quasi-isolated trap states enabled by the strong plasmon electric field, effectively extending the lifetime of hot electrons, and increase the probability of electron re-excitation by another low-energy photon.

Another *in situ* characterization methodology is electron spin resonance (ESR), also known as electron paramagnetic resonance. This technique stems from magnetic resonance principles and can detect the transition of unpaired electrons when exposed to a magnetic field [160]. Due to its remarkable ability to detect low concentrations, reaching approximately 10^−12^ mol, *in situ* ESR spectroscopy finds its primary application in tracking the formation of radicals on catalyst surfaces during reactions. This, in turn, helps identify potential reaction pathways and mechanisms. ESR has been used to investigate the CO_2_ reaction mechanisms on plasmonic catalysts [107], [165], [166], demonstrating the involvement of hot electrons in generating TNIs or reactive oxygen vacancies, which promote the activation of CO_2_.

#### 3.1.3 Imaging technique

**Figure 8:**
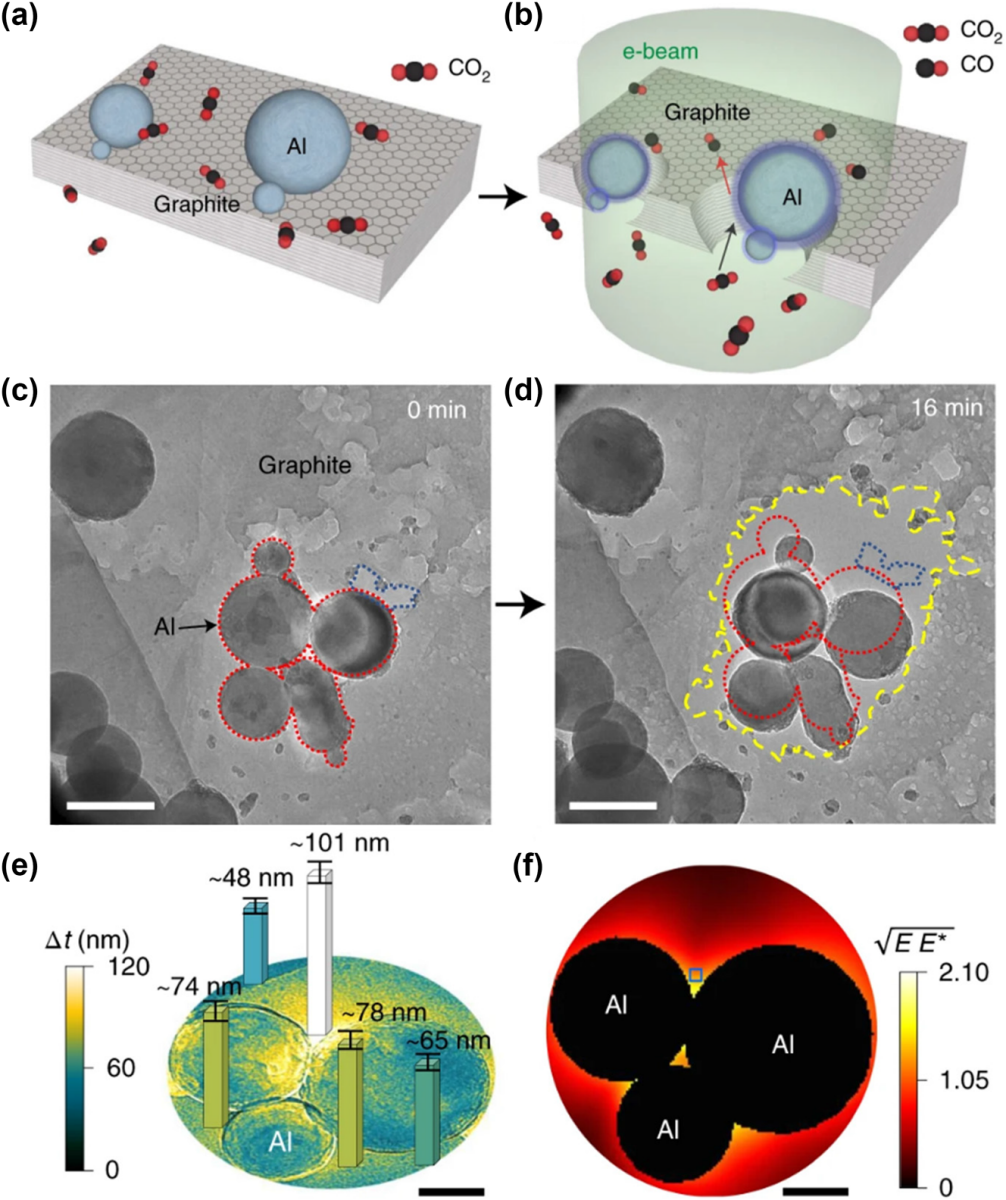
*In-situ* visualization of CO_2_ reduction to CO. (a, b) Schematic of Al NPs on a graphite flake, without and with electron beam illumination, respectively. (c, d) Time-resolved TEM images showing etching of graphite in a CO_2_ partial pressure of ∼50 Pa with a cluster of Al NPs. (e) Carbon depletion map representing the change of carbon thickness after the electron beam illumination. (f) Field distribution of the coupled Al NPs. Reproduced with permission from [167]. Copyright 2021, Springer Nature.

Unfortunately, the spectroscopic techniques discussed above do not offer spatial information. In order to visually capture a chemical reaction with nanoscale accuracy and assess the role of near-fields, Wang and colleagues employed *in situ* environmental scanning transmission electron microscope (ESTEM) coupled with a gas chromatography-mass spectroscopy setup [167]. They exploited the finely focused and highly energetic electron beam to excite LSPRs in Al nanoparticles placed on graphite flakes. This enabled them to observe the endothermic reduction of CO_2_ to CO by carbon at room temperature, a process commonly known as the reverse Boudouard reaction (CO_2_(g) + C(s) → 2CO(g)). The process is schematically shown in Figure 8a and b. Under illumination, the plasmon near-fields induced by the Al NPs drive the reaction, leading to the consumption of the graphite flakes underneath, as shown in the TEM images at different time steps in Fig. 8c and d. Through the quantification of etched graphite near the nanoparticles in a CO_2_ environment, they were able to determine the reaction rate. Simultaneously, they were able to measure both the temperature and spatial pattern of LSPR modes using electron energy loss spectroscopy (EELS). The researchers concluded that a near-field process facilitated by the Al NPs drove the observed reaction of CO_2_ with carbon. This was further confirmed by measuring the change of the thickness of the graphite flakes before and after (Figure 8(e)) electron beam illumination and comparing it with the simulated field distribution (Figure 8(f)). As expected, they found a correlation between the strength of the coupled near-fields and the amount of etched graphite. These results are in line with previous *in situ* ESTEM studies on dehydrogenation reaction on plasmonic Au-PdH_*x*_, which demonstrated the role of plasmonic near-fields in altering the active site location of hydride nucleation in PdH_*x*_ [168], [169]. This innovative approach enables precise spatial resolution of LSPR modes distribution on a nanometric scale, as well as the dynamic behaviour of plasmonic nanoparticles with millisecond precision. As a result, its scale-up holds significant promise in the field of photocatalysis.

In summary, *in situ* measurements have been used in conjunction with theoretical computations to investigate reactions mechanisms, resulting in important insights insights. In doing so, studies have uncovered the role of intermediates, and hot electrons, and provided evidence that plasmonic photocatalysts can help direct the reaction towards certain pathways. However, more work is needed to fully understand how plasmonic catalysts can enable selectivity.

### 3.2 Ensemble measurements

The overarching goal in this field is to achieve efficient CO_2_ conversion with high reaction yield to specific products, though a stable and scalable device.

In this section, we will focus on the most recent ensemble experimental results for CO_2_ reduction and will help the reader to identify trends and limitations of these systems. This section does not aim to be an exhaustive summary, but rather a synthesis of the seminal works and the current state-of-the-art, which have achieved the highest efficiency, selectivity and production of C_2+_ species. Table 1 provides a summary of various plasmonic catalysts along with their performance and experimental parameters. For detailed reviews on the materials aspect, we refer the reader to the excellent works in ref [15], [170], [171], [172].

**Table 1:** Summary of various plasmon photocatalytic systems for CO_2_ reduction ordered by decreasing product yield.

Catalyst	Main product	Minor products	Yield main product	Select., %	Illumination condition	Temp., °C	Notes/other	Ref.
Black Au–Ni	CO	CH_4_	2464 mmol g^−1^ h^−1^	95 %	Xe lamp (2.77 W cm^−2^)	223	Yield is normalized only to g_Ni_. Gas phase, flow reactor. Gas ratio CO_2_ : H_2_ = 10. Isotopic ^13^C labelling performed	[8]
Au–Ag_8_Cu_1_ alloy	CO	CH_4_	CO 1468.1 mmol g^−1^ h^−1^ CH4 398.9 mmol g^−1^ h^−1^	∼78 %	300 W Xe lamp (3.7 W cm^−2^)	310	Gas phase + 20 µL of H_2_O. Batch reactor. 50 % H_2_/50 % CO_2_ feed. Selectivity was computed. Isotopic ^13^C labelling performed	[107]
Ni_3_N	CO	CH_4_	1212 mmol g^−1^ h^−1^	99 %	Xenon lamp, 400–1100 nm, 3.006 W cm^−2^	199	Gas phase, flow reactor. Gas ratio CO_2_ : H_2_ = 10, 20. Isotopic ^13^C labelling performed	[173]
Au-grafted Ce_0.95_Ru_0.05_O_2_	CH4	CO	473 mmol g^−1^ h^−1^	∼100 %	Xe lamp (1.6/5.3 W cm^−2^)	340	Gas phase, flow reactor. 4 % H_2_, 1 % CO_2_ and 95 % Ar. Isotopic ^18^O labelling performed	[9]
Quantum-sized Au NPs	CO	CH_4_	4730 µmol g^−1^ h^−1^	∼100 %	LED@420 nm (73 mW cm^−2^)	200	Gas phase + 20 µL of H_2_O. Batch reactor. Isotopic ^13^C and H_2_^18^O labelling performed	[154]
Au@Pd NPs in UiO-66-NH_2_ MOF	CO	CH_4_	3737 µmol g^−1^ h^−1^	∼80 %	300 W Xe lamp	150	Gas phase, batch reactor Gas ratio CO_2_ : H_2_ = 3.	[174]
AuCu NPs on SrTiO_3_/TiO_2_ nanotube	CO	CH_2_, C_2_H_4_, C_2_H_6_, C_3_H_6_	3770 µmol g^−1^ h^−1^	∼83 %	300 W Xe lamp	–	Gas phase, batch reactor. Tot hydrocarbons yield 725 μmol g^−1^ h^−1.^ Isotopic ^13^C labelling performed	[175]
Au@ZIF-67	CH_3_OH	C_2_H_5_OH	2500 µmol g^−1^ h^−1^	∼95 %	Solar simulator (150 mW cm^−2^)	–	Aqueous solution. Ethanol yield is ∼480 μmol g^−1^ h^−1^	[176]
Au–Ag NPs/TIO_2_	CO	CH_4_, C_2_H_4_, C_2_H_6_, CH_3_OH, C_3_H_8_	1813 µmol g^−1^ h^−1^	∼97	Vis: Xe lamp (20 mW cm^−2^) UV: Hg lamp (150 mWcm^−2^)	–	Gas phase, batch reactor. Gas ratio CO_2_ : H_2_ = 1	[177]
Au NPs/TiO_2_	CO	CH_4_, CH_3_OH, C_2_H_4_, C_2_H_6_, C_3_H_6_, C_3_H_8_	1237 µmol g^−1^ h^−1^	–	Xe lamp (10 mWcm^−2^)	100	Gas phase, batch reactor. Gas ratio CO_2_ : H_2_ = 1	[178]
Au NPs/TiO_2_	CO	CH_4_, C_2_H_4_, C_2_H_6_, C_3_H_6_	1223 µmol g^−1^ h^−1^	98.9	Vis: solar simulator (100 mWcm^−2^) UV: Hg lamp (150 mWcm^−2^)	100	Gas phase, flow reactor Gas ratio CO_2_ : H_2_ = 1	[179]
Ag NPs/TiO_2_ NW	CO	CH_4_, CH_3_OH	983 µmol g^−1^ h^−1^	98	Hg lamp (20 mW cm^−2^)	100	Gas phase, flow reactor Gas ratio CO_2_ : H_2_ = 1	[180]
Au rod@CuPd_2_	CH_4_	C_2_H_4_, C_2_H_6_	550 µmol g^−1^ h^−1^	∼100 %	Xe lamp (400 mW cm^−2^)	100	CO_2_-saturated solution containing Au rod@CuPd_2_. Isotopic ^13^C labelling performed	[164]
Au/TiO_2_	CO	CH_4_	429 µmol g^−1^ h^−1^	98 %	Solar simulator (1.44 W cm^−2^)	150	Yield is normalized only to g_Au_. Pressure = 3.6 bar	[181]
Al@Cu_2_O	CO	CH_4_	360 µmol cm^−2^ h^−1^	∼100 %	Supercontinuum fibre laser (10 W cm^−2^)	180	Gas phase, flow reactor. CO_2_/H_2_ feed	[182]
Cu–Ru	CH_4_	CO	∼275 µmol g^−1^ s^−1^	>99 %	Supercontinuum fibre laser (19.2 W cm^−2^)	750	Gas phase, flow reactor. Gas ratio CO_2_ : CH_4_ = 1. Isotopic ^13^C labelling performed	[139]
Ag/AgClB	CH_3_CHO	CO, CH_4_, C_2_H_4_	209 µmol g^−1^ h^−1^	96.9	500 W Xenon lamp with AM 1.5G filter (100 mW cm^−2^)	25	Liquid phase with NaHCO_3_ and triethylamine (TEA). Batch reactor. Isotopic ^13^C labelling performed	[155]
Au/m-ZnO	C_2_H_6_	CH_4_, CO	27 µmol g^−1^ h^−1^	∼65 %	300 W Xe lamp (595 mW)	25	Gas phase + 20 µL of H_2_O. Batch reactor.	[165]
Au/TiO2	CH_4_	C_2_H_6_, HCHO CH_3_OH	15 μmol m^−2^ h^−1a^	–	UV @ 254 nm (20 mW cm^−2^)	75	Gas phase + H_2_O, batch reactor. ^a^Yield was computed.	[183]
AgCu–TiO_2_ nanotubes	C_2_H_6_	CH_4_	14.5 µmol g^−1^ h^−1^	60.7 %	Solar simulator (100 mW cm^−2^)	50	Gas phase + few droplets of H_2_O. Batch reactor Isotopic ^13^C labelling performed	[184]
Au–Pd on TiO_2_	CH_4_	C_2_H_4_, C_2_H_6_, CO	14.3 µmol g^−1^ h^−1a^	85 %^a^	300 W Xe lamp (853 mW cm^−2^)	40	^a^Yield and selectivity are for total hydrocarbon. Gas-phase + 100 µL of H_2_O. Batch reactor. Isotopic ^13^C labelling performed	[185]
Au NPs + EMIM-BF_4_	CH_4_	C_2_H_2_, C_2_H_4_, C_3_H_6_, C_3_H_8_	4.8 NP^−1^ h^−1^	50 %^a^	532 nm laser (1 W cm^−2^)	48	^a^Selectivity is for C_2+_ products. Batch reactor. Aqueous solution + EMIM-BF_4_ Isotopic ^13^C labelling performed	[4]
Au NPs	CH_4_	C_2_H_6_	0.68 NP^−1^ h^−1^	–	Xe lamp (300 mW cm^−2^)	47	Batch reactor. Aqueous solution (water + IPA)	[157]
Rh/Al_2_O_3_	CH_4_	CO	0.1–7 µmol cm^−2^ s^−1^	90–95 %	UV: @365 nm (3 W cm^−2^)	300–600	Gas phase, continuous flow. Gas ratio CO_2_ : H_2_ = 1:3.1. Isotopic D_2_ labelling performed	[10]
	CH4	CO	0.05–0.9 µmol cm^−2^ s^−1^		Blue LED (2.4 W cm^−2^)	300–600		
Au/TiO_2−*x*_	CH_4_	C_2_H_6_	2.5 μmol g^−1^ h^−1^	60 %	300W Xe lamp	–	Gas phase + 400 µL of H_2_O. Batch reactor.	[186]
Cu–TiO_2_	CH_4_	–	124 ppm cm^−2^ h^−1^	–	AM1.5 illumination (100 mW/cm^2^)		Gas phase, batch reactor with wet CO_2_	

There is a wide variability in terms of reaction yield, product distribution and selectivity, which can be largely attributed to the different experimental conditions (liquid-phase vs gas-phase, broadband vs monochromatic excitation, batch vs flow reaction) resulting from a lack of standard procedures in the field [187]. Variations in the emission spectra of the light source, reaction conditions and reactor configurations have been shown to lead significant changes in the product amounts and distribution for the same reaction and catalyst [187], [188], [189], [190]. There are also discrepancies in the units used to report the yield: while most works report the product amount in moles per gram per hour (µmol g^−1^ h^−1^), others normalize to the illuminated area (µmol cm^−2^ h^−1^) or use parts-per-million (g^−1^ h^−1^), and some use the concept of turnover number. To allow for comparisons between experimental findings, it is essential to report experimental parameters including reactor design, power and emission spectra of light source, gaseous and liquid volume of the reactor, reaction temperature and pressure, amount of catalyst used and its specifications (porosity, active surface area, absorption), sacrificial agents (if present), stability, turnover number and frequency. For these reasons, the field of plasmonic photocatalysis would benefit from the implementation of standard procedures for experimental conditions and data reporting.

Table 1 shows that most reported yields are small, on the order of few tens or hundreds of μmol g^−1^ h^−1^, and the studies with the highest yields, in the range of mol g^−1^ h^−1^, produce single carbon atom products. Despite this, these works provide fundamental understanding of reaction mechanisms, demonstration of C_2+_ products and proof of concept of selectivity in CO_2_ photoreduction.

**Figure 9:**
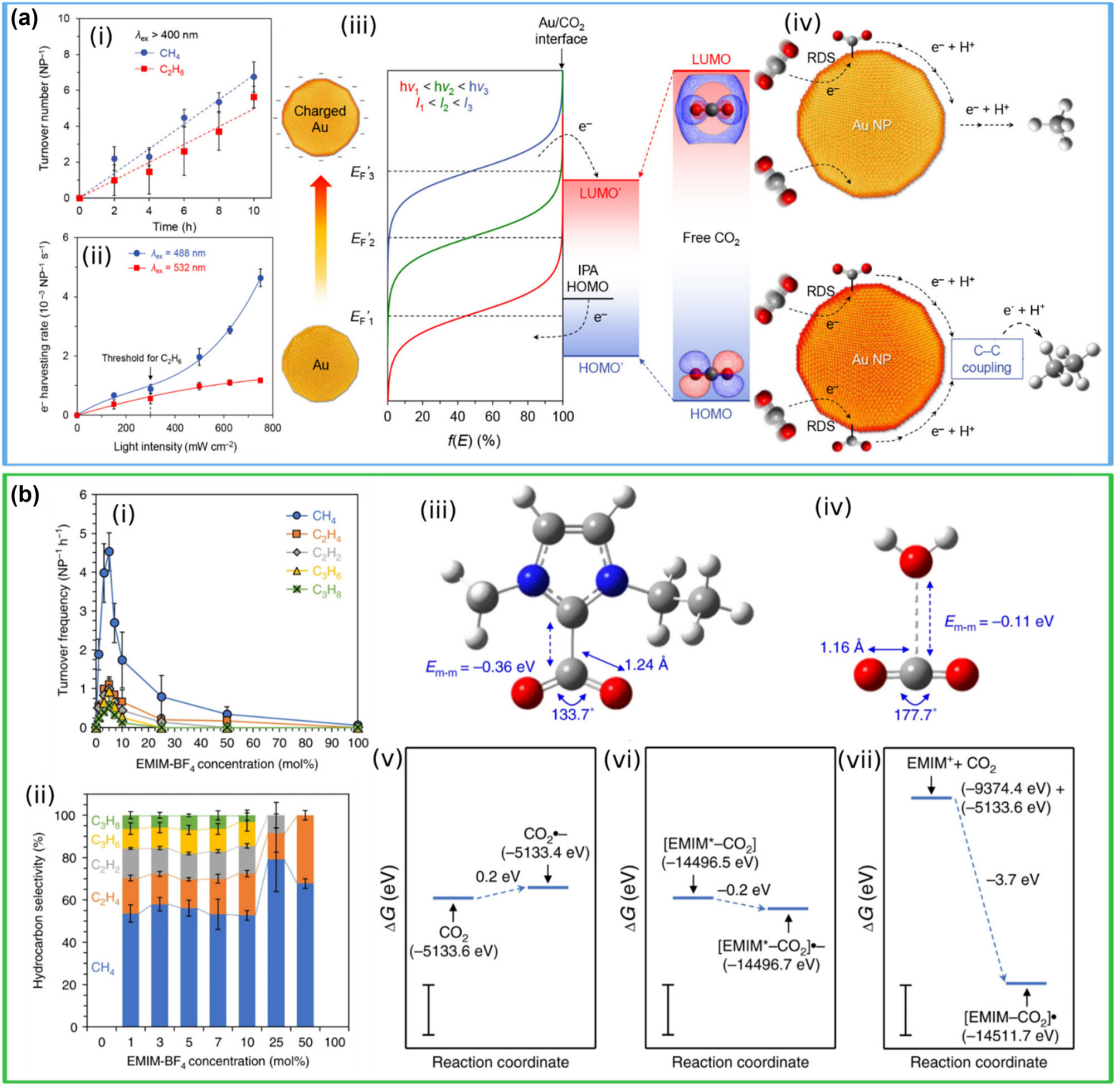
Plasmon-driven CO_2_ reduction to C_2+_ species. (a-i) Turnover number as a function of the reaction time for the CH_4_ and C_2_H_6_ products. (a-ii) Rate of hot electrons harvesting for the two products as a function of the light intensity. (a-iii, iv) Schematic of the plasmon-assisted CO_2_ reduction mechanism and formation of C_1_ and C_2_ products. (b-i, ii) Turnover frequency and selectivity of the produced hydrocarbons as a function of the EMIM-BF4 concentration, respectively. (b-iii, iv) DFT geometries of [EMIM*-CO_2_] and [H_2_O–CO_2_] complexes, respectively. C, H, O and N atoms are grey, white, red and blue, respectively. (b-v, vi, vii) Computed free energy, ∆*G*, for adding 1e^−^ to CO_2_, to [EMIM*-CO_2_] to CO_2_ in the presence of EMIM+. The free energy of each species is indicated in parentheses. Scale bars are 1 eV in length. (a) Reproduced with permission from ref [157]. Copyright 2018, American Chemical Society. (b) Reproduced with permission from [4]. Copyright 2019, Springer Nature.

For instance, the work by Yu et al. demonstrated the kinetically challenging multi-electron, multi-proton, plasmonic-assisted photocatalytic CO_2_ reduction to methane (CH_4_) and ethane (C_2_H_6_) [157]. Using dispersed Au NPs in water with isopropanol (IPA) as a sacrificial hole scavenger, the authors achieved turnover numbers (TONs) of ∼6.8 and ∼5.6 NP^−1^ after 10 h of illumination, for CH_4_ and C_2_H_6_, respectively (Figure 9(a, i)). Additionally, they showed that product formation is dependent on the excitation wavelength and intensity (Figure 9(a, ii)): under 532 nm illumination, corresponding to the LSPR of Au NPs, CH_4_ is the only product and hence the electron harvesting rate is linearly dependent on the laser intensity (Figure 9(a, ii)). However, when illuminating at *λ*_ex_ = 488 nm, corresponding to the interband transitions of Au, C_2_H_6_ was formed when light intensities reached (300 mW cm^−2^, as shown by the superlinear dependence of the electron harvesting rate on light intensity in Figure 9(a, ii). In line with previous works [191], [192], the authors proposed that hybridization between the metal and molecule states decreases the molecular HOMO–LUMO gap (Figure 9(a, iii–iv)). Simultaneously, CW illumination cathodically polarizes the NP, making it a source of energetic electrons for CO_2_ activation, thus forming a radical ion intermediate, CO_2_^•–^. This highly active TNI then leads to the formation of CH_4_. Under high light intensity and interband excitation, more than one electron transfer can take place within the surface residence time of adsorbed CO_2_, resulting in the simultaneous activation of two CO_2_ adsorbates, which can couple and promote the formation of C_2_H_6_.

The same group have also shown the direct synthesis of C_1_–C_3_ hydrocarbons using Au NPs illuminated by a green (532 nm) CW laser (Figure 9(b, i-ii)). While the main product is CH_4_, they also measured ethylene (C_2_H_4_), acetylene (C_2_H_2_), propane (C_3_H_8_) and propene (C_3_H_6_), with selectivity for C_2+_ hydrocarbons of up to 50 %. These results were enabled by using an ionic liquid – 1-ethyl-3-methylimidazolium tetrafluoroborate (EMIM-BF_4_) – to stabilize charged intermediates and promote electron transfer at the interface between Au NPs and CO_2_. Results of DFT computations (Figure 9(b, iii–vii)) showed that CO_2_ strongly interacts with EMIM-BF4, forming a complex [EMIM*-CO_2_] and binding to the C2 atom of the imidazole ring with an energy of −0.36 eV, much stronger than the interaction of an H_2_O molecule and CO_2_ (Figure 9(b, iii–iv)). The ionic liquid also promotes the bending and thus activation of CO_2_, converging to a OCO angle of 133.7°. Through a comparative analysis (Figure 9(b, v–vii)) of the free energy required to transfer one electron to the different species participating the CO_2_ reduction (CO_2_, [EMIM*-CO_2_] and CO_2_ in the presence of EMIM+), the authors have proposed that EMIM-BF_4_ can promote the transfer of photogenerated electrons from the Au NP to adsorbed CO_2_, which is otherwise a major kinetic bottleneck in the photocatalytic reduction process.

To the best of our knowledge, this is the only work that has obtained C_3_ products using photocatalysis with pure (only metals) plasmonic materials. Traces of C_3_ products (<10 μmol g^−1^ s^−1^) have been reported when using plasmonic NPs (Au, Ag) as a co-catalysts in hybrid plasmon-semiconductor systems (TiO_2_, SrTiO_3_/TiO_2_) [175], [177], [178]. In these experiments, the proposed mechanism is that hot electrons (holes) generated from the plasmonic NPs are injected in the conduction (valence) band of the semiconductors, improving charge separation, and promoting CO_2_ reduction and H_2_ oxidation, respectively. Despite these promising results for the realization of valuable fuels and chemicals from CO_2_ and (green) H_2_, the yields and selectivity for C_3_ compounds are still very low.

**Figure 10:**
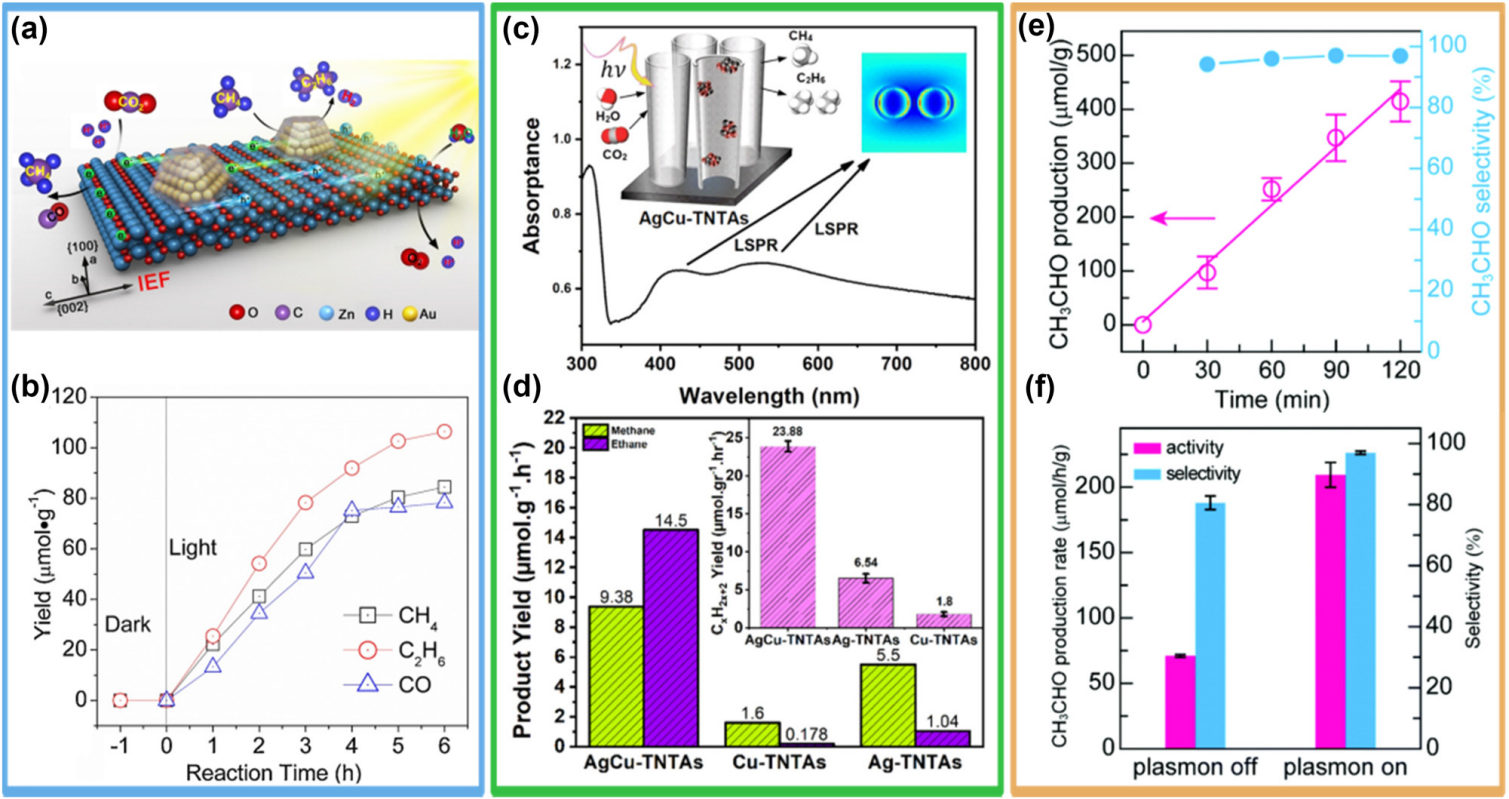
CO_2_ photoreduction to C_2+_ species with hybrid plasmon-semiconductor materials. (a) Schematic representation of Au/ZnO nanosheets for CO_2_ photoreduction and (b) product evolution under light illumination. (c) Absorptance of TiO2 nanotubes arrays decorated with AgCu NPs along with its schematic representation and field enhancement at the LSPR. (d) Product distribution of TiO_2_ nanotube arrays decorated with different plasmonic NPs. (e) Photocatalytic CH_3_CHO production activity and selectivity of the Janus Ag/AgCl_0.79_Br_0.21_ nanostructures as a function of the reaction time. (f) Effect of light irradiation on the product yield and selectivity. (a–b) Reproduced with permission from [165]. Copyrights 2019, Elsevier. (c–d) Reproduced with permission from [184]. Copyright 2021, American Chemical Society (e–f) Reproduced with permission from [155]. Copyrights 2021, Royal Society of Chemistry.

Recently, high selectivity towards C_2_ products and moderate reaction rates were successfully achieved by exploring different combinations of hybrid plasmonic-semiconductors photocatalysts [155], [165], [184], [185]. Zhao et al. [165] used porous ZnO nanosheets decorated with noble metals – Au, Ag and Pd – (Figure 10(a)) and performed a comparative analysis on the CO_2_ photoreduction performance. The reaction was carried out in the gas-phase and under solar (*λ* > 320 nm) illumination, using 20 µL of water as a source of protons, without any other sacrificial agent. Interestingly, the choice of plasmon metal drastically influenced the product distribution: Ag achieved 86 % selectivity towards CO with a yield of ∼25 μmol g^−1^ h^−1^ (remain product was CH_4_ at ∼4 μmol g^−1^ h^−1^), conversely Pd achieved 85 % selectivity towards CH_4_ with a yield of ∼18 μmol g^−1^ h^−1^ (remain product was CO ∼3.5 μmol g^−1^ h^−1^), while Au surprisingly achieved 65 % selectivity towards C_2_H_6_ with a production rate of ∼27 μmol g^−1^ h^−1^ (remaining products were CH_4_ and CO, both at ∼20 μmol g^−1^ h^−1^). This study demonstrated that CO_2_ photoreduction with Ag and Pd results only in C_1_ species, while Au preferentially leads to production of C_2_H_6_ which is formed by converting CH_4_ via a dehydrogenative coupling mechanism (2CH_4_ → C_2_H_6_ + H_2_) [193]. The reaction was further investigated by DFT calculations, spin trapping electron paramagnetic spectroscopy and photo-electrochemical measurements. The authors suggested that the introduction of plasmon materials have a twofold purpose: (i) the plasmon near-fields couple and enhance the spontaneously generated inner electric field formed between stacked ZnO nanosheets, resulting in a more efficient charge separation and increased carrier lifetime, and (ii) enable plasmon resonant energy transfer across the ZnO bandgap via interband transition.

The effect of different metals on the production of C_2_ species was also investigated by Vahidzadeh et al. [184]. Here, the authors tested densely packed TiO_2_ nanotubes arrays (TNTA) decorated with Ag, Cu and AgCu alloys NPs towards the visible (AM 1.5G 1-sun illumination) gas-phase photoreduction of CO_2_ without using any sacrificial agent or hole scavengers (Figure 10(c)). They found that the addition of large sized (80–200 nm) NPs increases both the efficiency and selectivity of CO_2_ photoreduction. As shown in Figure 10(d), the AgCu-TNTA exhibited higher product yields (14.5 μmol g^−1^ h^−1^ of C_2_H_6_ and 9.38 μmol g^−1^ h^−1^ of CH_4_) and higher selectivity (60.7 % towards ethane) for C_2_ products compared to TNTAs decorated with monometallic Ag or Cu NPs. The authors attributed the superior performance towards C_2_H_6_ to cooperative effects of (i) the high plasmonic hot-spots density, (ii) the asymmetric charge distribution generated from closely packed NPs which decreases adsorbate–adsorbate repulsion and improves C–C coupling and (iii) increased lifetime of hot electrons injected over the Schottky barrier formed at the metal–semiconductor interface, although a rigorous mechanistic study was missing. As hot electrons transfer is widely accepted to be more efficient in small nanoparticles [17], [62], [194], the high performance achieved by using large plasmonic NPs (>80 nm) suggests that in these systems near-field enhanced mechanisms are more significant than hot electrons transfer.

While most of the studies have detected gas-phase products, converting CO_2_ into liquid (or solid) compounds holds significant practical importance because of their higher energy density and ease of storage and transportation [195], [196]. In a recent study [155], broadband Janus silver/ternary silver halide (Ag/AgClBr) nanostructures were shown to achieve a record-high 96.9 % selectivity towards acetaldehyde (CH_3_CHO) with a generation rate of 209 μmol g^−1^ h^−1^ under solar illumination, while just traces of other species (CO, CH_4_ and C_2_H_4_) were observed (Figure 10(e)). The photocatalysts were dispersed into an aqueous solution containing 0.1 M NaHCO_3_ and triethylamine as a hole scavenger. This is a surprising result as the formation of CH_3_CHO is an energy intensive reaction, which requires transfer of 10 electrons. The authors also performed several control experiments, including the ^13^CO_2_ isotope labelling, to confirm that acetaldehyde was effectively being produced by the CO_2_ reduction on the catalyst. To investigate the mechanism, the authors performed the reaction under full-solar illumination (plasmon-on state in Figure 10(f)) and *λ*_ex_ > 420 nm, to prevent the excitation of the Ag LSPR at 350 nm (plasmon-off state in Figure 10(f)). Upon solar illumination (plasmon-on), the authors detected more than a threefold increase in the reaction yield along with an improved selectivity compared to the plasmon-off state. They attributed the increase in performance to a synergistic effect of enhanced near-fields and hot electron transfer, which promotes the system to an excited potential energy surface, improving selectivity and CH_3_CHO production rate.

**Figure 11:**
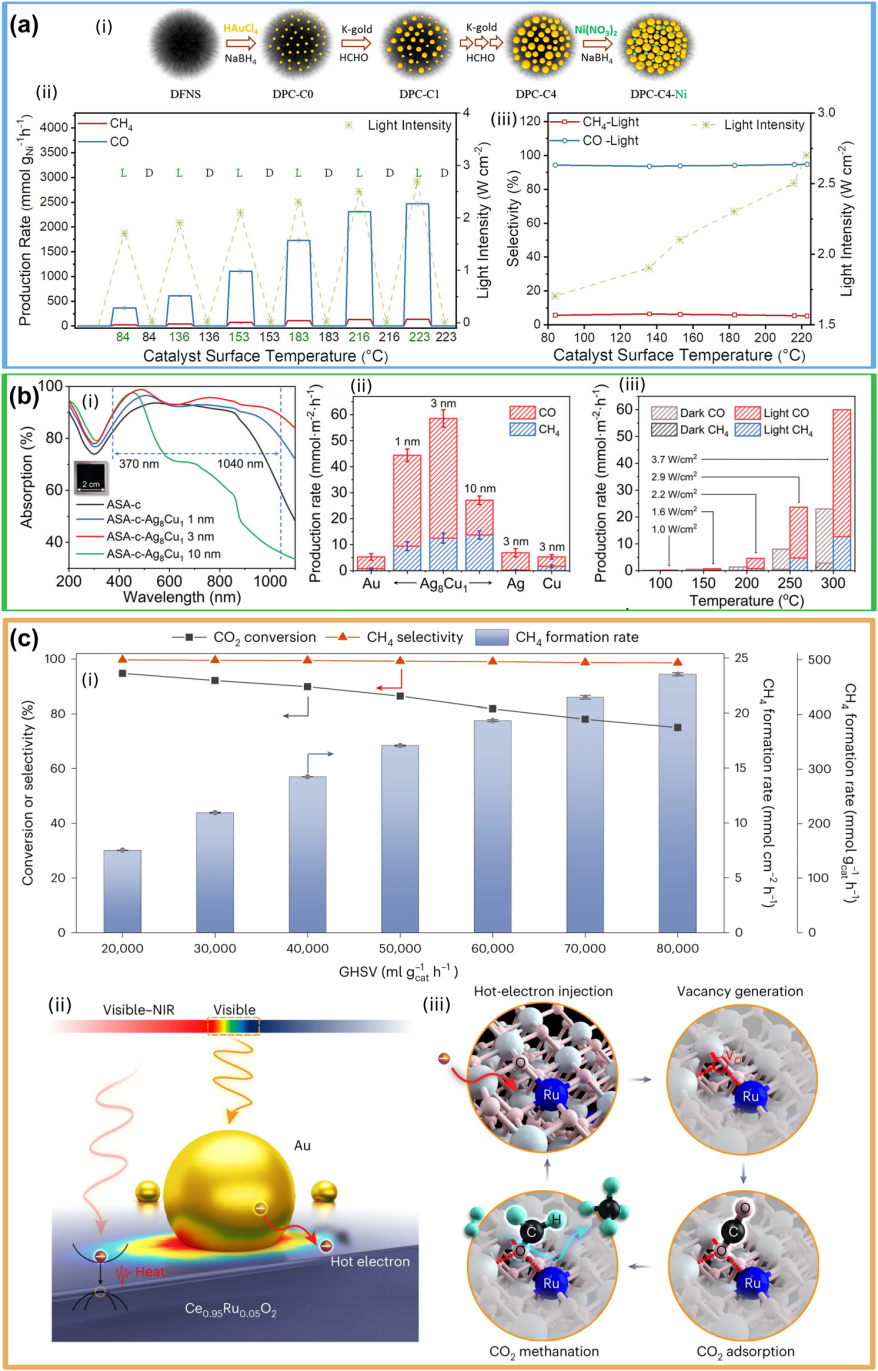
High performing CO_2_ to C_1_ products photocatalysis. (a-i) schematic of the synthesis of Ni-laden black-gold dendritic colloidosomes. (a-ii) Plasmonic CO_2_ hydrogenation reaction yield upon multiple light-on light-off cycles, without any external heating. (a-iii) Products selectivity measured at different light intensities. (b-i) Absorption profiles of different stacked plasmonic metamaterials. (b-ii) Production rate of CO and CH_4_ for plasmonic CO_2_ hydrogenation and (b-iii) comparison between reaction rates obtained in dark and light conditions for the different metamaterials. (c-i) Schematic of the cooperative photochemical and photothermal effects in the plasmon-enhanced CO_2_ methanation process (c-ii). CO_2_ conversion, CH4 selectivity and production rate of the CO_2_ methanation on Au_0.1_/Ce_0.95_Ru_0.05_O_2_ as a function of the gas hourly space velocity (GHSV) using a concentrated reactant gas. (a) Reproduced with permission from [8]. Copyright 2023, American Chemical Society. (b) Reproduced with permission from [107]. Copyright 2022, Wiley-VCH GmbH. (c) Reproduced with permission from [9]. Copyright 2023, Springer Nature.

In order to improve the productivity of plasmon-enhanced CO_2_ conversion, an inspiring strategy is to use broadband absorbers. Recently, dendritic plasmonic colloidosomes of Au loaded with nickel, DPC-C4-Ni, (Figure 11(a, i)) achieved extremely high performances for the gas-phase CO_2_ hydrogenation reaction [8]. The authors have taken advantage of the broadband absorption and high surface area of the composite structure, along with the enhanced catalytic activity of Ni, to achieve a record-high CO production rate of 2464 ± 40 mmol g_Ni_^−1^ h^−1^ with 95 % selectivity (Figure 11, (ii–iii)) and stability up to 100 h. The reaction was carried out in a flow reactor at atmospheric pressure and without external heating. It is worth noting that such high rate is atypical for photocatalysis – the amount of products is generally in the tens or hundreds of μmol g^−1^ h^−1^ (see Table 1) – and it is comparable with reaction rates obtained from electrocatalysis. Upon illumination, the plasmon relaxation process described in Section 2 leads to a catalyst surface temperature of 223 °C. Interestingly, a comparison of the reaction yield under illumination and in the dark (with external heat provided to reach the same temperature as under illumination) revealed a 9-fold increase in activity in light as compared to the dark and demonstrating the importance of non-thermal effects. *In situ* DRIFT spectroscopy showed that CO is weakly bonded to the active Ni sites, inferring that its desorption is efficient, thus restricting further hydrogenation to CH_4_ and leading to ∼95 % CO selectivity. Meanwhile, findings from kinetic isotope effect and ultrafast transient absorption spectroscopy demonstrated that hot electrons generated from the gold NPs are indirectly transferred to the catalytically active Ni sites and then participate in the reaction, similarly to antenna-reactor systems [101], [139], [164].

Reaction rates of the same order of magnitude were also achieved by using a novel non-noble metal plasmonic material for CO formation. By exploiting the plasmonic properties of hydroxy-terminated Ni_3_N nanosheets, Singh et al. [173] demonstrated efficient CO_2_ hydrogenation reaction, obtaining high CO production rate of 1212 mmol g^−1^ h^−1^ with ∼99 % CO selectivity, under visible illumination, in a flow reactor and without any sacrificial agent. Nickel is widely used in electrocatalysis to achieve efficient water splitting [197], [198], and these results shown that it also has high catalytic activity and affinity towards CO production, suggesting that Ni and metal nitrides could be used to develop efficient plasmonic photocatalysts.

The CO_2_ hydrogenation reaction was also recently studied by Shao et al. [107] with broadband periodic metamaterials. Contrary to the previous works, this research intentionally exploited the strongly localized near-fields and hot electrons generated from the plasmonic elements to achieve intense localized temperature increase on the device to enable the photo-thermal CO_2_ conversion. The authors used a multistep template-assisted colloidal lithography technique to fabricate a plasmonic metamaterial consisting of stacked Au_film_–SiO_2_–Au_patterned_-SiO_2_-Ag_8_Cu (ASA-c-Ag_8_Cu) layered structure. Here, the Au_patterned_ consists of tapered triangular arrays and the Au film acts as a rear mirror and is necessary to achieve >80 % absorption across the vis-NIR (Figure 11(b, i)) by increasing internal light scattering, SiO_2_ improved thermal stability by preventing heat deformation of the Au_patterned_ and finally Ag_8_Cu provides the catalytically active site. Thanks to the enhanced light absorption, localized near-fields and accumulation of hot electrons, this complex structure achieves surface temperatures as high as 310 °C under full-spectrum light illumination at 3.7 W cm^−2^. This led to reaction yields of 398.9 and 1468.1 mmol g^−1^ h^−1^ for CH_4_ and CO, respectively. Figure 11(b, ii–iii) reports these production rates in units of mmol m^−2^ h^−2^. As shown in Figure 11(b-iii), the reaction rates in the dark at the same temperature are substantially lower than those under light irradiation, indicating the importance of plasmonic excitation, in line with previous works [8], [173]. Further results obtained from electrodynamic simulations and *in situ* XPS and *in situ* electron paramagnetic resonance (EPR), suggested that all the plasmon relaxation processes synergistically contribute to promoting CO_2_ activation and reducing the energy barrier for CO_2_ hydrogenation. Strong localized near-fields promote hot electron generation and transfer from Au to the catalytically active Ag_8_Cu and synergistically enhance the photothermal effect, leading to higher catalytic activities.

A similar strategy was recently used to promote efficient photothermal CO_2_ methanation on Au/Ce_0.95_Ru_0.05_O_2_ solid-solution catalyst [9]. By utilizing visible-near-infrared light irradiation (350–2500 nm, 5.3 W cm^−2^) without any supplementary heating, this catalyst demonstrated a high CH_4_ formation rate of 473 mmol g^−1^ h^−1^ with ∼100 % selectivity and single-pass CO_2_ conversion rate of ∼75 % (Figure 10), surpassing the activity of conventional bandgap-excitation photocatalysts and approaching the thermodynamic limit. This was accomplished in a continuous flow reactor aimed at simulating the industrial CO_2_ methanation process, by using the same gas composition (72 % H_2_, 18 % CO_2_, balance Ar) and similar gas hourly space velocity (GHSV). The latter is an important parameter in CO_2_ methanation studies as it affects the reaction rate and selectivity of the process and represent the volumetric flow rate of the gas feed per unit volume of the catalyst bed per hour. Upon careful investigation, the authors found that the reaction was initiated by photothermal effects and enhanced by the plasmon excitation. As shown in Figure 11(c-ii, iii), upon absorption of vis-NIR light Ce_0.95_Ru_0.05_O_2_ generates heat, which increases the catalysts surface temperature up to ∼340 °C, while visible excitation of the LSPR generates hot electrons, which are then injected into Ce_0.95_Ru_0.05_O_2_ creating surface oxygen vacancies (V_O_) near the ruthenium sites. These Ru-V_O_ are the catalytically active centres and accelerate the CO_2_ methanation process, by facilitating CO_2_ adsorption and H_2_ dissociation. The V_O_ exhibit similarities to transient negative ions (TNIs), as both involve charged sites affecting chemical reactions; however, TNIs typically refer to short-lived species that play a role in reaction intermediates, while V_O_ are structural defects influencing catalytic behaviour [199]. Despite the fact that the surface temperature reaches up to ∼340 °C, the bulk temperature of the system is just ∼50 °C, which is notably lower than the conditions required for industrial reactors (200–550 °C with pressures of 1–100 bar). Along with the high reaction rate and excellent selectivity, this study indicates the potential advantages of the LSPR-enhanced photothermal CO_2_ methanation reaction over conventional energy-intensive thermal systems which, if applied at industrial scale, could offer a clean and sustainable solution for storing intermittent renewable energy [200].

## 4 Outlook and perspective

To conclude, this review provides a comprehensive overview of the recent advancements in plasmonic photocatalysis for CO_2_ reduction, focusing on the key mechanisms, challenges and trends. We delved into the fundamental principles underlying the interactions between plasmonic nanoparticles, light and molecular species, elucidating how these interactions can be harnessed to drive efficient energy transfer processes during heterogeneous photocatalysis. We then explored the various experimental strategies that have been used to differentiate between the different competing and/or cooperating effects. Lastly, we summarized the latest cutting-edge research efforts focused on the mechanistic understanding of reaction pathways as well as at achieving high performance in CO_2_ reduction. We hope to have provided factual demonstration that more often than not, synergistic plasmonic energy transfer mechanisms promote the reaction and increase the efficiency. Near-fields can significantly increase molecular absorption; hot carrier transfer can initiate reactions and plasmonic photothermal heat generation can increase catalytic activity, all of which can result in enhanced photochemical reactions. Throughout our examination, it has become evident that plasmonic photocatalysis holds great promise in addressing the pressing global challenges of converting CO_2_ into valuable sources of clean energy. However, as we navigate within this field, several challenges and questions persist, including the optimization of plasmonic catalysts, the scalability of these processes and the long-term stability of plasmonic nanomaterials. The path forward will require interdisciplinary collaboration, innovation in materials design and the integration of cutting-edge techniques, such as machine learning, to unlock the full potential of plasmonic photocatalysis.

Recently, the emergence of the new sub-field of vibropolaronic chemistry [201] has shown that strong coupling of molecules with optical modes – where photons are exchanged faster than competing dissipative processes – can lead to new hybrid light–matter polaritonic states, which can modify the potential energy surface. Coupling of vibrational modes with the vacuum field of Fabry–Perot cavity or optical cavity modes [44], [202] can thus provide a new degree of freedom in modulating chemical reactions, thus opening a new avenue for the field of heterogeneous catalyst.

Additionally, understanding the role of hot holes is essential for optimizing solar-driven CO_2_ reduction strategies and to improve the activity for C_2+_ species. Better utilization of hot holes could help provide additional protons necessary to produce value-added hydrocarbons and alcohols and, therefore, improve yields. In particular, exploring efficient metal–semiconductor interfaces could improve charge separation and extend the lifetime of photogenerated hot electrons and holes, ultimately increasing reaction rates and selectivity.

Simultaneously, the integration of machine learning algorithms in the field of heterogeneous photocatalysis [203], [204] could help boost the field. The drastic and exponential growth of these approaches, along with their ability in dealing with big data, holds great promise for propelling the field of plasmonic and heterogeneous photocatalysis into new frontiers of discovery and application. By exploiting the power of data-driven algorithms, it is expected that there will be an important shift from DFT methodologies to machine learning approaches, which could enable to gain deeper insights into complex reaction mechanisms, accelerate materials discovery and optimize reaction conditions.

Leveraging the remarkable reaction yields and selectivity towards C_1_ species achieved by broadband plasmonic materials coupled with semiconductors or other catalytically active species, we anticipate that broadband and highly absorptive plasmon catalysts will play a pivotal role in harnessing and converting electromagnetic energy from sunlight towards the production of valuable C_2+_ species with high yield and selectivity. As we stand on the threshold of a new era in materials science and catalysis, the marriage of plasmonic photocatalysis with surface science, advances in characterization techniques and computational methodologies offers an exciting and dynamic path forward, promising breakthroughs that may revolutionize energy conversion, environmental remediation and other critical areas of science and technology.
